# The small nonstructural protein NP1 of human bocavirus 1 directly interacts with Ku70 and RPA70 and facilitates viral DNA replication

**DOI:** 10.1371/journal.ppat.1010578

**Published:** 2022-06-02

**Authors:** Kang Ning, Zekun Wang, Fang Cheng, Ziying Yan, Jianming Qiu

**Affiliations:** 1 Department of Microbiology, Molecular Genetics and Immunology, University of Kansas Medical Center, Kansas City, Kansas, United States of America; 2 Department of Anatomy and Cell Biology, University of Iowa, Iowa City, Iowa, United States of America; Stony Brook University, UNITED STATES

## Abstract

Human bocavirus 1 (HBoV1), a member of the genus *Bocaparvovirus* of the family *Parvoviridae*, causes acute respiratory tract infections in young children. Well-differentiated pseudostratified human airway epithelium cultured at an air-liquid interface (HAE-ALI) is an ideal *in vitro* culture model to study HBoV1 infection. Unique to other parvoviruses, bocaparvoviruses express a small nonstructured protein NP1 of ~25 kDa from an open reading frame (ORF) in the center of the viral genome. NP1 plays an important role in viral DNA replication and pre-mRNA processing. In this study, we performed an affinity purification assay to identify HBoV1 NP1-inteacting proteins. We identified that Ku70 and RPA70 directly interact with the NP1 at a high binding affinity, characterized with an equilibrium dissociation constant (K_D_) of 95 nM and 122 nM, respectively. Furthermore, we mapped the key NP1-interacting domains of Ku70 at aa266-439 and of RPA70 at aa181-422. Following a dominant negative strategy, we revealed that the interactions of Ku70 and RPA70 with NP1 play a significant role in HBoV1 DNA replication not only in an *in vitro* viral DNA replication assay but also in HBoV1-infected HAE-ALI cultures. Collectively, our study revealed a novel mechanism by which HBoV1 NP1 enhances viral DNA replication through its direct interactions with Ku70 and RPA70.

## Author summary

HBoV1, an autonomously replicating human parvovirus, causes acute respiratory tract infections in young children. HBoV1 infects and productively replicates in non-dividing (well-differentiated) cells of HAE-ALI. HBoV1 replication induces a DNA damage response and activates DNA repair, which drive viral genome amplification. In this report, we identified that NP1, a small nonstructural protein encoded in the center of the viral genome, directly interacts with Ku70 and RPA70 at a high affinity. The key interaction domains of Ku70 and RPA70 were characterized and confirmed by the inhibition of HBoV1 viral DNA replication both in a newly established *in vitro* viral DNA replication assay and in HBoV1-infected HAE-ALI. Thus, our study demonstrated that the interactions of NP1 with Ku70 and RPA70 enhance HBoV1 DNA replication, highlighting the essential role of the small nonstructural protein in parvoviral DNA replication and the importance of the DNA repair factors in viral DNA replication.

## Introduction

Human bocavirus 1 (HBoV1) belongs to *Primate bocaparvovirus 1* in the genus *Bocaparvovirus* of the *Parvoviridae* family [1–3]. HBoV1 was first identified in 2005 by a large-scale virus screening of nasopharyngeal specimens of children with respiratory illness [1], and was later confirmed to be an emerging human pathogen that causes lower respiratory tract infections in young children worldwide [2,4–13]. After infection, the replication of HBoV1 in airways was detected in high viral loads during the acute phase and persisted at a low viral load for several months.

HBoV1 expresses one large nonstructural protein (NS1), four small nonstructural proteins (NS2, NS3, NS4, and NP1), one small noncoding RNA (bocavirus-encoded small RNA, BocaSR), and three viral capsid proteins (VP1, VP2, and VP3) from a single precursor mRNA (pre-mRNA) via alternative splicing [14–18]. NS1, NP1, and BocaSR are essential for DNA replication of HBoV1. Among the nonstructural proteins, the expression of the 25-kDa NP1 is a unique feature of bocaparvoviruses. In the family *Parvoviridae*, only the members of the genus *Bocaparvovirus* express NP1 from a unique open reading frame (ORF) located in the middle of the genome [2,19,20]. Minute virus of canines (MVC) NP1 was the first bocaparvoviral NP1 identified to play an important role in viral DNA replication and viral pre-mRNA processing through interacting with cleavage and polyadenylation specificity factor 6 (CPSF6) [20–22]. HBoV1 NP1 also interacts with CPSF6 and regulates polyadenylation of capsid protein-encoding mRNA [23]. Moreover, the transport of HBoV1 NP1 to the nucleus is escorted by CPSF6 [23]. Importantly, HBoV1 NP1 colocalizes with replicating viral DNA in the viral DNA replication centers [24].

Autonomously replicating parvoviruses, including HBoV1, are able to hijack the host DNA damage and repair machinery to facilitate their genome replication [25–32]. *In vitro*, HBoV1 infects well-differentiated (polarized) human airway epithelium (HAE) cultured at an air-liquid interface (HAE-ALI) [30,33–37]. HBoV1 infection of HAE-ALI or DNA replication in proliferating HEK293 cells triggers a DNA damage response (DDR) with the activation of phosphatidylinositol 3-kinase–related kinases (PI3KKs), including ataxia telangiectasia-mutated kinase (ATM), ATM and Rad3-related kinase (ATR), and DNA-dependent protein kinase catalytic subunit (DNA-PKcs) [30,31]. The Ku and replication protein A (RPA) complexes are indispensable components during DDR induction and DNA damage repair [38–40]. The Ku complex, as a heterodimer of Ku70 and Ku80, binds to the ends of double-stranded DNA break (DSB) and recruits DNA-PKcs, which is required for the non-homologous end joining (NHEJ) pathway of DNA repair [38,41,42]. As a DNA binding protein, Ku70 contains 3 different domains: the von Willebrand A (vWA) domain at the N-terminus, the Core domain in the center, and the SAP (AF-A/B, Acinus, and PIAS motifs) domain at the C-terminus [38]. Notably, the Ku complex has 3’→ 5’ DNA helicase activities [43]. The RPA complex is a heterotrimeric protein complex composed of RPA70, RPA32, and RPA14 [44,45]. Functioning as single-stranded DNA (ssDNA) binding proteins, the RPA complex binds to ssDNA, protects ssDNA from nucleolytic damages and hairpin formation until the DNA replication or repair is processed [45,46]. Within the RPA70 subunit, the primary protein binding domains are localized in the N-terminus and the middle AB domain, and the C terminus mainly executes the heterotrimerization function with RPA32 and RPA14, although both AB domain and C-terminus also bind to ssDNA [44,45]. During parvovirus DNA replication, the RPA complex has to be recruited in proximity to the parvoviral DNA replication origin that is nicked by the large nonstructural proteins, NS1 or Rep78/68. In adeno-associated virus (AAV) replication, the interaction of Rep78/68 with RPA complex enhances binding and nicking of the Rep proteins at the origin [47]. For another parvovirus minute virus of mice (MVM), NS1 directly interacts with the RPA complex and involves extensive unwinding of the nicked origin [48]. We previously identified that HBoV1 NS1 interacts with Ku70 and RPA70 [49]; however, whether NS1 directly interacts with RPA70 is unknown.

In the current study, we expressed Flag-tagged HBoV1 NP1 (NP1^Flag^) to supplement the replication of the NP1-depleted HBoV1 full-length duplex DNA in HEK293 cells, by which an affinity pull-down assay was performed to identify cellular proteins that interact with NP1^Flag^. An amount of 77 proteins were addressed with > 14 unique reads by mass spectrometry (MS), among which Ku70 and RPA70 were further dissected to directly interact with NP1. We revealed that NS1 neither directly interacted with RPA70 nor with NP1. These findings have led to an HBoV1 DNA replication model in which the NP1 functions as a mediator to recruit the RPA and Ku complexes to the viral DNA replication complex to facilitate viral DNA replication.

## Results

### Affinity pull-down of HBoV1 NP1^Flag^ identified cellular proteins that interact with the NP1

**Fig 1:**
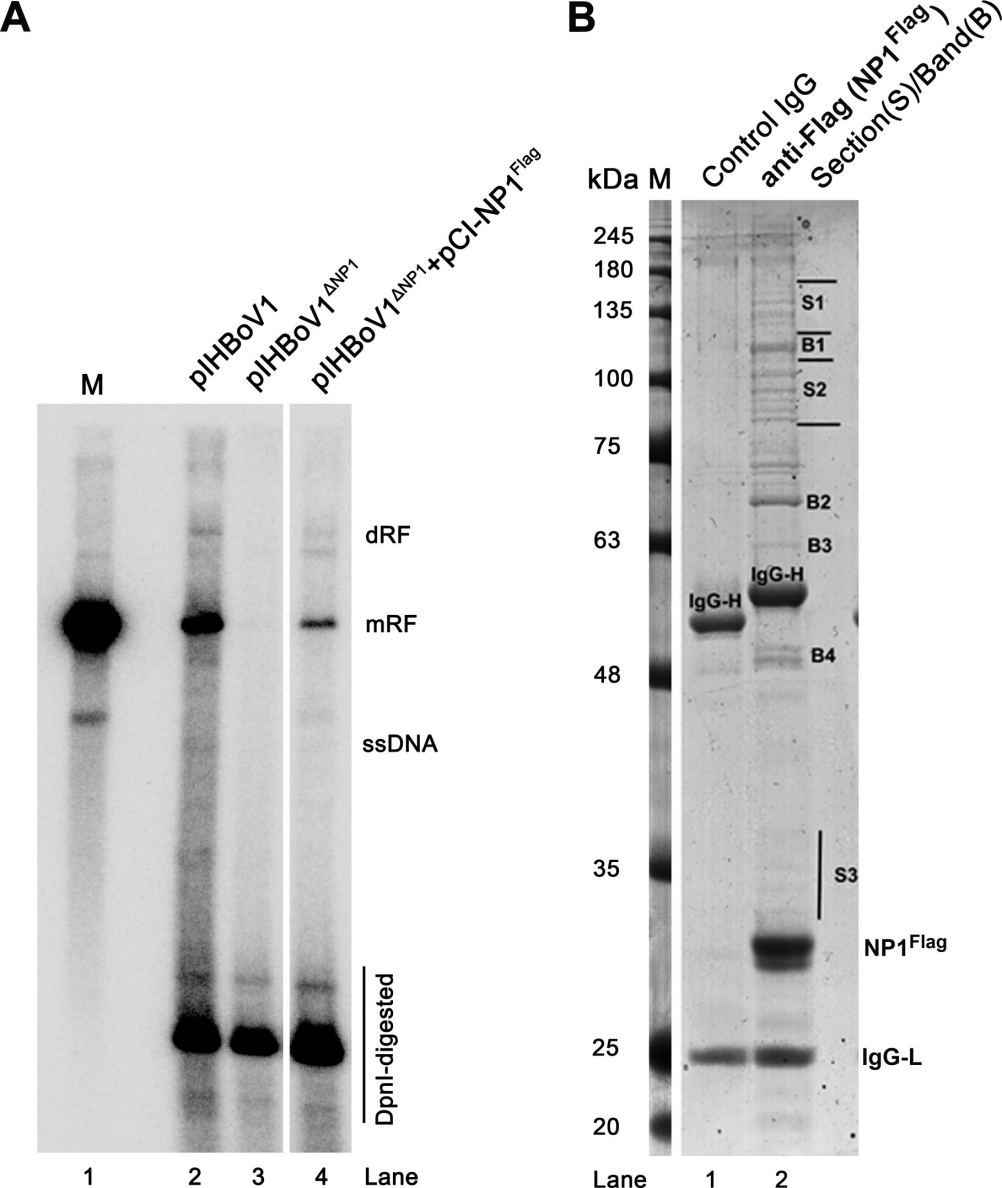
Affinity purification of NP1-interatcing cellular proteins. **(A) Southern blotting.** HEK293 cells were transfected with pIHBoV1, pIHBoV1^ΔNP1^, and pIHBoV1^ΔNP1^ plus pCI-NP1^Flag^, respectively. At 2 days post transfection, Hirt DNA were extracted in each transfection group and analyzed by Southern blotting using a full-length HBoV1 genome as a probe. HBoV1 duplex DNA excised from pIHBoV1 was used as a size marker (M) of ~5.5 kb (Lane 1). dRF, mRF, and ssDNA represent double, monomer replicative form DNA and single stranded DNA, respectively. DpnI digested DNA is indicated. **(B) Co-immunoprecipitation (IP) and SDS-(10%)PAGE.** HEK293 cells were transfected with pIHBoV1^ΔNP1^ and pCI-NP1^Flag^. At 2 days post transfection, cell lysates were immunoprecipitated with an anti-Flag (NP1^Flag^, lane 2) or control IgG (lane 1). Immunoprecipitated proteins were separated on SDS-10%PAGE, followed by Coomassie Brilliant Blue staining. Sections (S) 1–3 and Bands (B) 1–4 that uniquely appeared in the sample immunoprecipitated with anti-Flag (lane 2). M, molecule weight ladder.

**Fig 2:**
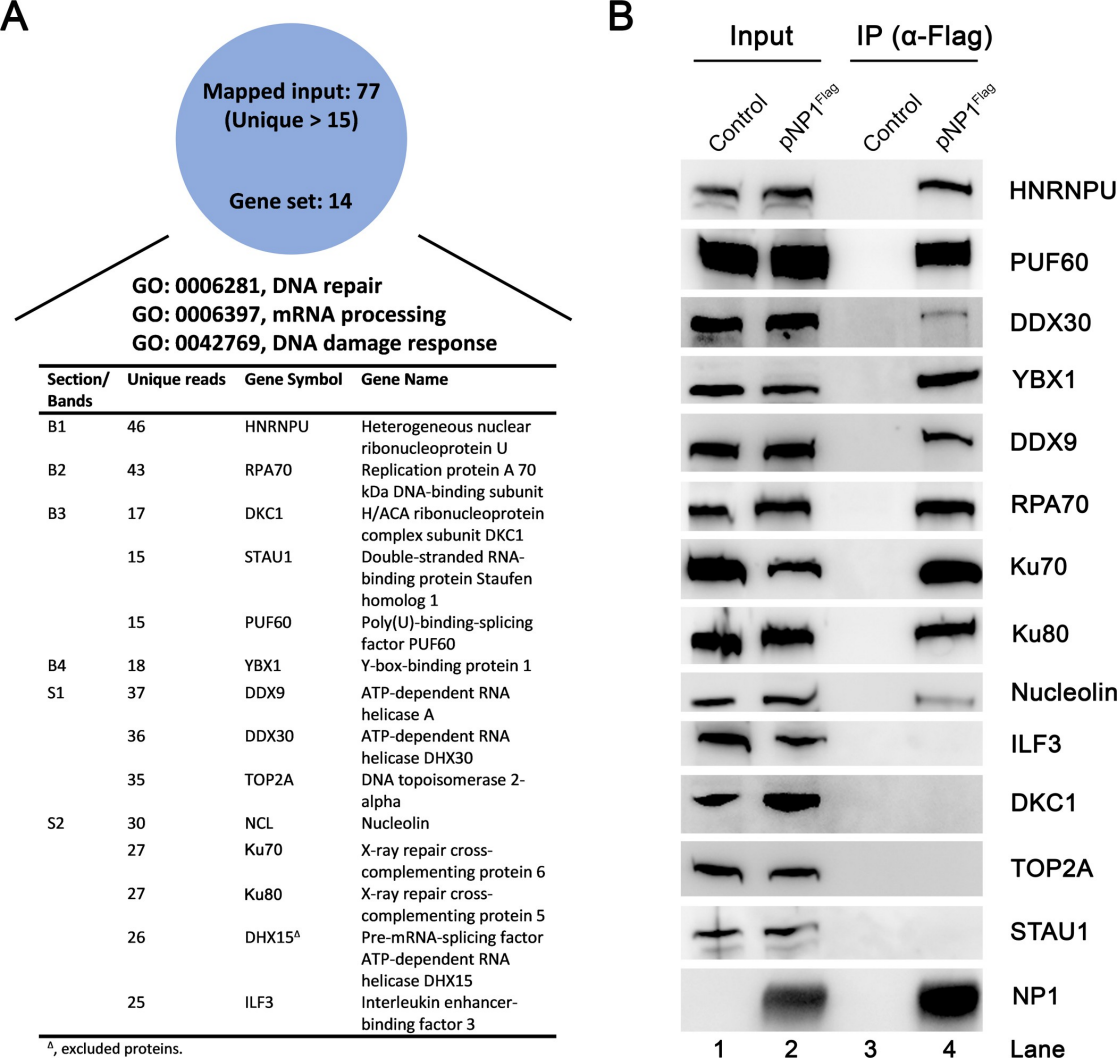
Confirmation of HBoV1 NP1-interacting proteins using co-immunoprecipitation (Co-IP) assay. HEK293 cells were transfected with pCI-NP1^Flag^ or pCI (as a vector control). At 48 h post-transfection, immunoprecipitation was performed with anti-Flag-conjugated agarose resin using Lysis buffer containing Benzonase, followed by Western blotting. **(A) Gene Ontology (GO) analysis.** The GO analysis of HBoV1 NP1-interacting proteins with unique peptide reads of over 14 by mass spectrometry (MS) was performed using Metascape (http://metascape.org/) and categorized by their biological processing functions. A summary of key NP1-interacting host proteins that have functions in DNA repair, mRNA processing, and DNA damage response are shown. Proteins identified in each section/band pulled down with anti-Flag (NP1^Flag^) are shown with gene symbols and protein names, respectively. **(B) Co-IP.** Selected NP1 interacting proteins involved in DNA repair, mRNA processing, and DNA damage response were confirmed by Co-IP assays. Proteins detected by respective antibodies (against the 13 hits in panel **A**) are indicated at the right of each image. 10% of the cell lysates were loaded as input controls (lanes 1&2).

We previously identified HBoV1 NP1 plays an important role in HBoV1 DNA replication [15,35] and regulation of viral mRNA processing [16,23]. To further address the function of NP1 in viral DNA replication, we performed an affinity pull-down assay using NP1^Flag^ as a bait to identify cellular proteins that interact with NP1 during HBoV1 DNA replication (**Fig 1A**). HEK293 cells were co-transfected with pCI-NP1^Flag^ and pIHBoV1^ΔNP1^ that has NP1 knocked out [35], enabling the isolation of proteins involved in the interaction with NP1 by anti-Flag affinity purification. Three major sections (S1-S3) and 4 bands (B1-B4) were uniquely presented in the lane of the proteins pulled down by anti-Flag, but not in the control lane, on the Coomassie blue stained sodium dodecyl sulfate-polyacrylamide gel electrophoresis (SDS-PAGE) gel (**Fig 1B**). Samples of the in-gel digestions of these sections and bands excised from the PAGE gel were used for mass spectrometry (MS) analysis. The MS results showed that 77 proteins were addressed with more than 14 unique reads (**S1 Table**). Gene Ontology (GO) term enrichment analysis revealed 14 proteins were involved in DDR, DNA repair, and mRNA processing (**Fig 2A**).

Next, we performed co-immunoprecipitation (co-IP) to confirm the interaction of NP1 with these 14 selected proteins. To this end, HEK293 cells were transfected with pCI-NP1^Flag^ or mock transfected with an empty vector (pCI) as control. At 3 days post-transfection, the cell lysates were incubated with anti-Flag affinity beads for co-IP assay. Nuclease (Benzonase) was added in the binding buffer to eliminate any DNA or RNA. The Co-IP results showed that 9 proteins, including heterogeneous nuclear ribonucleoprotein U (HNRNPU), poly (U) binding splicing factor 60 (PUF60), DExH-box helicase 30 (DHX30/DDX30), Y-box binding protein 1 (YBX1), DHX9/DDX9, RPA70, ATP-dependent DNA helicase II 70 KDa (Ku70), Ku80 and nucleolin were pulled down by anti-Flag-conjugated beads (**Fig 2B**). Notably, DHX15/DDX15 has been previously confirmed to interact with NP1 [23]. The other 4 proteins, ILF3, DKC1, TOP2A, and STAU1, were not confirmed by Co-IP in the presence of nuclease, suggesting their interactions with NP1 are mediated by DNA or RNA.

Taken together, we identified 9 cellular proteins, HNRNPU, PUF60, DDX30, YBX1, DDX9, RPA70, Ku70, Ku80 and nucleolin specifically interacted with NP1 without the involvement of DNA/RNA mediation.

### Ku70 but not Ku80 directly interacts with HBoV1 NP1

**Fig 3:**
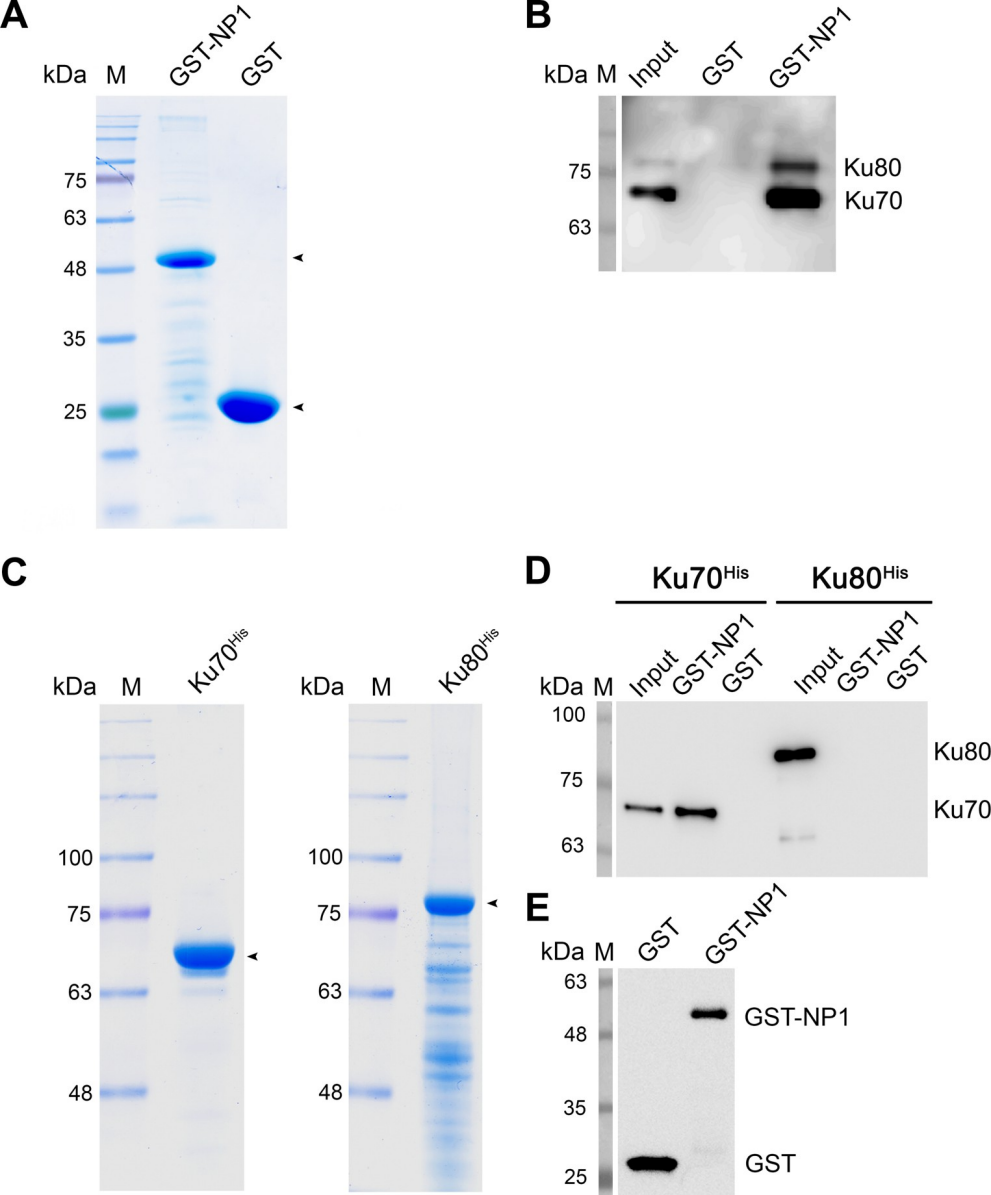
NP1 directly interacts with Ku70 but not Ku80. **(A) Purification of HBoV1 GST-NP1 and GST proteins.** HBoV1 GST-NP1 and GST protein were purified as described in Materials and Methods. The eluted GST-NP1 and GST proteins from peaked fractions were separated on an SDS-(4–20%)PAGE gel and stained with Coomassie brilliant blue. **(B) *In vitro* pull-down assay of Ku70/80 heterodimer.** 4 μg of purified GST-NP1 was used as bait to pull down 4 μg of the prey protein, purified His-tagged Ku70/80 heterodimer (#CT018-H07B, SinoBiological, Wayne, PA), using Glutathione agarose. 4 μg of GST served as a negative control. Proteins pulled down by the Glutathione agarose were separated on SDS-PAGE gel and blotted with anti-His (**B**). ~0.4 μg of Ku heterodimer was used as an input. **(C-E) Purification and *in vitro* pull-down of Ku70**^**His**^ **and Ku80**^**His**^. Ku70^His^ and Ku80^His^ proteins were purified and analyzed on an SDS-(10%)PAGE gel (C). 4 μg of purified GST-NP1 and negative control GST protein were used as bait to pull down 4 μg of the prey protein, purified Ku70^His^ and Ku80^His^, respectively, using Glutathione agarose. ~0.4 μg of the bait and prey proteins were loaded as inputs. Purified proteins were denoted by arrowheads and the pulldown proteins were analyzed by Western blotting using anti-His for Ku70^His^ and Ku80^His^ (D) or using ant-GST for GST-NP1 and GST pulled down by glutathione agarose (as a control) (E).

During HBoV1 infection, Ku70 specifically interacts with HBoV1 NS1 and plays a role in viral DNA replication [49]. To further understand the role of the Ku complex in HBoV1 DNA replication, glutathione S-transferase (GST)-tagged NP1 proteins were produced (**Fig 3A**). Used as a bait, GST-NP1 pulled down the Ku complex (Ku70/80^His^ heterodimer), but not the GST alone (**Fig 3B**). To further address which component of the Ku heterodimer interacts with NP1, we purified hexahistidine (His)-tagged Ku70^His^ and Ku80^His^ proteins (**Fig 3C**) and used them to perform a pull-down assay. The results showed that Ku70^His^, but not Ku80^His^, interacted with GST-NP1 (**Fig 3D and 3E**). The interaction was independent on any residual nucleic acids in the reaction, as nuclease (benzonase) addition in the binding buffer, as well as incorporation of ethidium bromide (**S1 Fig**), did not prevent the interaction. The interaction was also independent of the fused GST, as a small Strep tagged NP1 (NP1^Strep^) pulled down Ku70 and the Ku complex (**S2 Fig**). Importantly, NP1 binding to Ku70 monomer did not affect the stability of Ku70 (**S3 Fig**).

Collectively, we confirmed that Ku70 of the Ku heterodimer directly interacts with HBoV1 NP1.

### The β-barrel domain of Ku70 is the key domain that interacts with HBoV1 NP1

**Fig 4:**
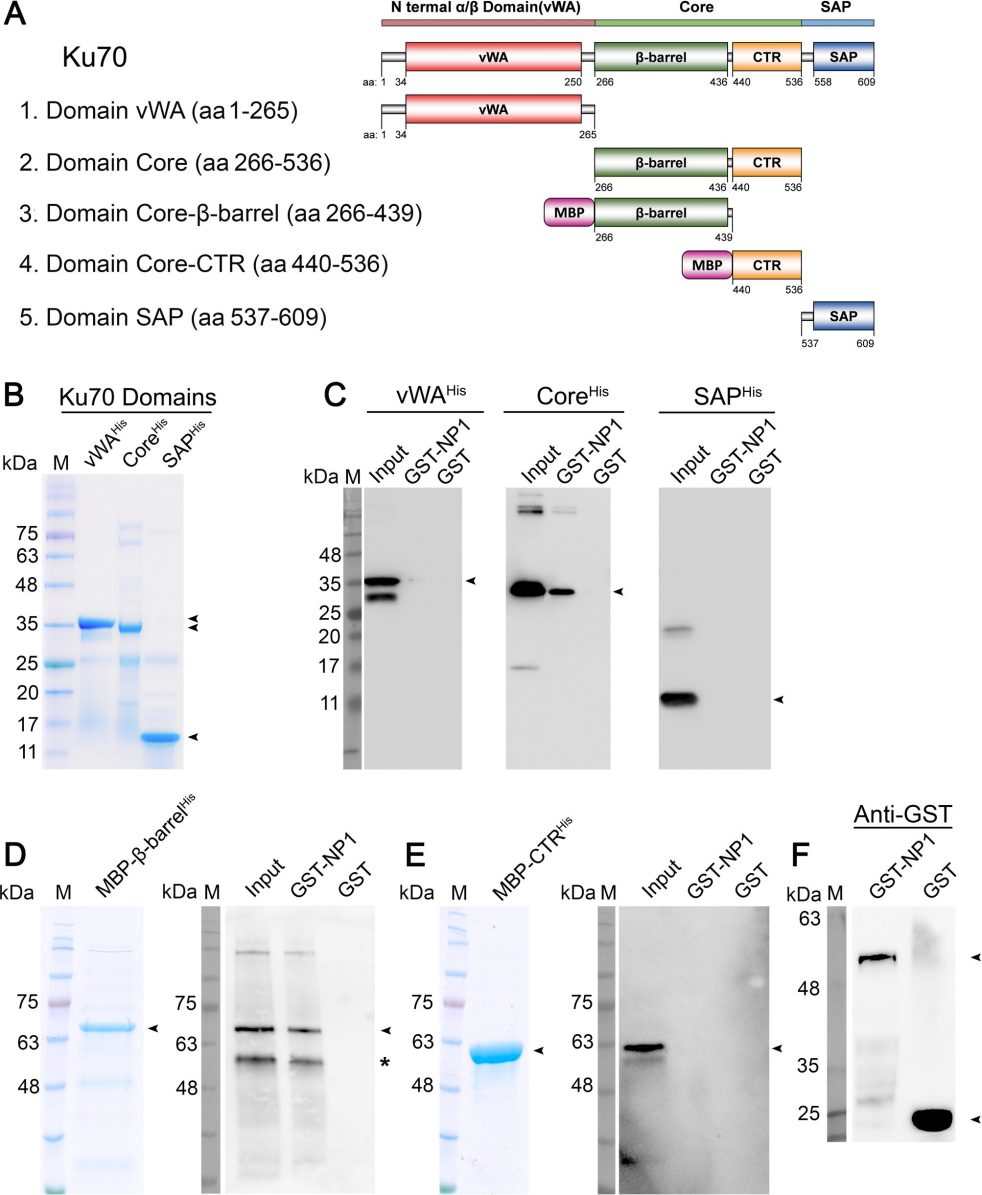
Identification of Ku70 domain that directly interacts with HBoV1 NP1. **(A) A diagram of Ku70 domains.** The N-terminal α/β domain (vWA) is shown in red, β-barrel and CTR domains within the middle Core domain in green and yellow, and C-terminal domain (SAP) in blue. Linkers are shown in silver and the fused Maltose-binding protein (MBP) in pink as shown. **(B&C) Purification of Ku70 domains and *in vitro* pull-down assay of His-tagged proteins.** Purified Ku70 truncated domains were analyzed on SDS-4-20%PAGE gel (B). vWAHis, Core^His^, and SAP^His^ were incubated with GST-NP1 and GST, respectively, followed by addition of Glutathione agarose. Pulldown proteins were analyzed by Western blotting with an anti-His antibody (C). **(D-F) *In vitro* pulldown of MBP fused protein.** 4 μg of purified GST-NP1 and GST were used as bait to pull down 4 μg of purified Ku70 truncated prey proteins, MBP-β-barrel^His^ (D, left) and MBP-CTR^His^ (E, left). Pulldown proteins were analyzed by Western blotting with an anti-His antibody (D&E, right) or with anti-GST (F). ~0.4 μg of prey proteins was used as input. Arrowheads denote purified proteins or detected domains. Arrowheads indicate the major detected protein bands and an asterisk indicates bands of degraded proteins.

As a DNA-binding protein, Ku70 contains 3 functional domains: the vWA domain at the N-terminus, the Core domain in the center and the SAP domain at the C-terminus [38] (**Fig 4A**). To dissect which domain interacts with NP1, we purified three His-tagged mini Ku70 proteins with one domain only [vWA^His^ (aa1-265), Core^His^ (aa266-536) and SAP^His^ (aa537-609)] (**Fig 4B**), and used them in the pull-down assays with GST-NP1 individually. The results showed GST-NP1 pulled down the Ku70-Core^His^, but not the Ku70-vWA^His^ or the Ku70-SAP^His^ (**Fig 4C**, GST-NP1), indicating that the Ku70-Core is the key NP1-interacting domain. As controls, none of these proteins interacted with GST protein (**Fig 4C**, GST). Furthermore, the Ku70-Core domain was dissected into the β-barrel (aa266-439) and CTR (aa440-536) subdomains. Due to the difficulties in purification of the two small domains in the monomeric form, we produced maltose-binding protein (MBP)-fused domains, MBP-β-barrel^His^ and MBP-CTR^His^, instead (**Fig 4D and 4E**). The pull-down assays showed GST-NP1 was able to pull down MBP-β-barrel^His^, but not MBP-CTR^His^ (**Fig 4D and 4E**, GST-NP1), indicating that the β-barrel domain of Ku70 is the key NP1-interacting domain. As controls, neither of these two proteins interacted with the GST protein (**Fig 4D and 4E**, GST), while the glutathione-agarose beads pulled down the GST proteins, as expected (**Fig 4F**).

**Fig 5:**
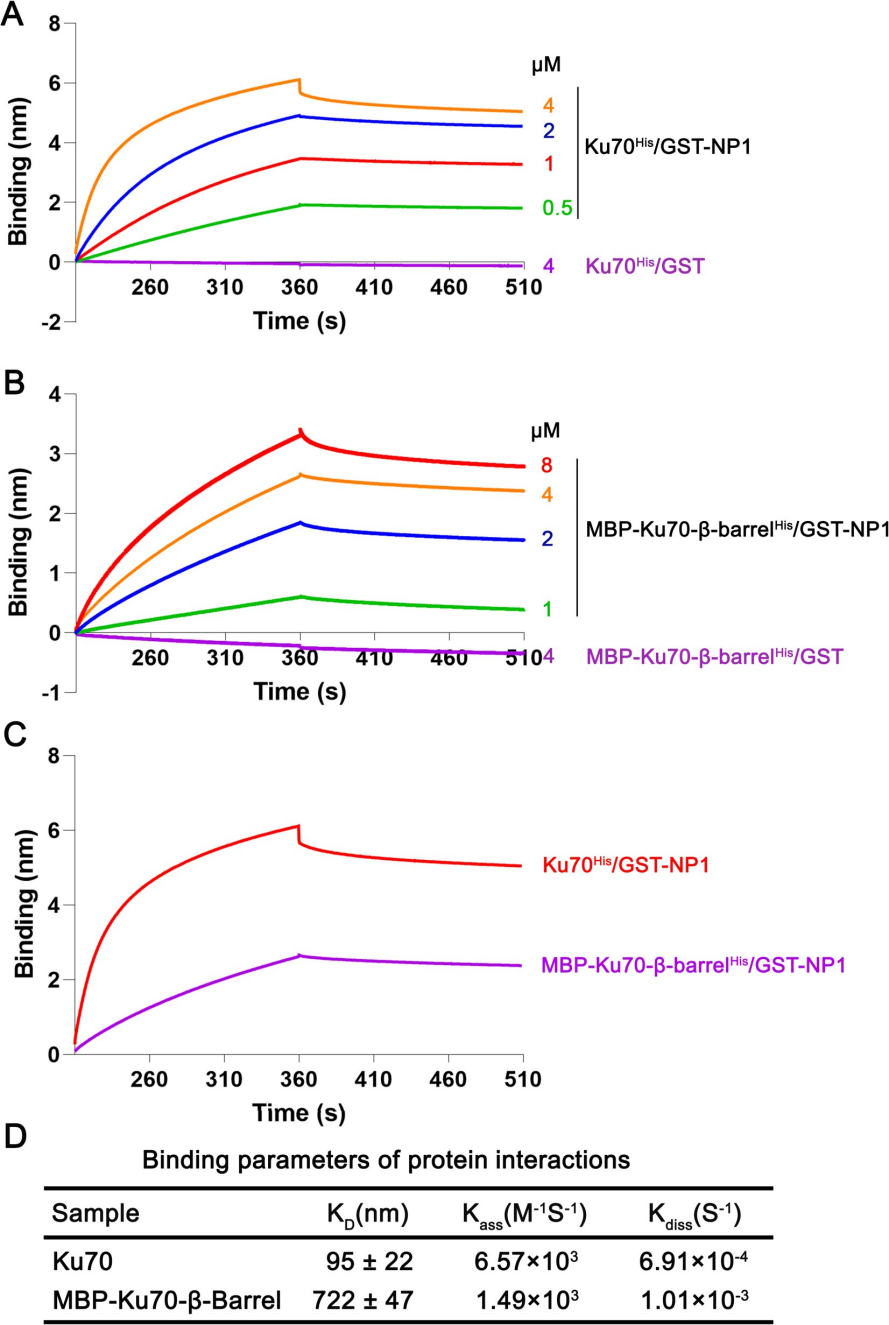
BLI analysis of the interaction kinetics between Ku70 and MBP-Ku70-β-barrel^His^ with HBoV1 NP1. **(A-B) Kinetic assays using Ni-NTA biosensors.** The binding kinetics of associations and dissociations of 4 μM GST-NP1 with (A) Ku70^His^ and (B) MBP-Ku70-β-barrel^His^ at different indicated concentrations. Binding kinetics of 4 μM GST with 4 μM Ku70^His^ or MBP-MBP-Ku70-β-barrel^His^ is shown as controls. **(C) Comparison of the binding affinities of NP1 to Ku70 and to Ku70-β-barrel.** The binding kinetics of association and dissociation of Ku70^His^ (4 μM) and MBP-Ku70-β-barrel^His^ (4 μM) with GST-NP1 (4 μM). **(D) Binding parameters of protein interactions.** Equilibrium dissociation constant K_D_ value represents the ratio of dissociation [K_diss_ (S^-1^)] and association [K_ass_ (M^-1^S^-1^)] computed from the real-time binding curves of GST-NP1 to Ku70^His^ or to MBP-Ku70-β-barrel^His^. The K_D_ values are shown with means ± standard deviations based on at least three repeated experiments.

We next used a Bio-Layer Interferometry (BLI) assay to determine the binding kinetics of Ku70 and its key binding domain β-barrel with NP1. Different concentrations of Ku70^His^ from 0.5 to 4 μM and MBP-β-barrel^His^ from 1 to 8 μM were loaded, respectively, to the assays for the association and dissociation with 4 μM GST-NP1. Results showed a significant shift in the association/dissociation curve in a concentration dependent manner (**Fig 5A and 5B**). As controls, 4 μM of both Ku70^His^ and MBP-Ku70-β-barrel^His^ showed negligible binding to 4 μM GST protein. Equilibrium dissociation constant (K_D_) was calculated from K_ass_ (M^-1^S^-1^) and K_diss_ (S^-1^) derived from the binding kinetics assays (**Fig 5D**). The K_D_ value of 95 nM indicated a robust binding of the Ku70 to NP1. The Ku70 β-barrel domain showed a decreased binding affinity (K_D_ = 722 nM) with GST-NP1, 7.6-fold lower compared with the full length Ku70, indicating that other domains maximize the binding capability. The binding kinetics of Ku70^His^ and Ku70-β-barrel^His^ with GST-NP1 was further compared at the same concentration of 4 μM in a single BLI assay, and the results confirmed the binding kinetics of GST-NP1 with MBP-Ku70-β-barrel^His^ but at a level poorer than that with Ku70^His^ (**Fig 5C**).

Taking these results together, BLI studies confirm the strong interaction of NP1 with Ku70. We determined that the Ku70 Core (aa266-536) is the key domain that interacts with the NP1 protein, and that the β-barrel domain (aa266-439) within the Ku70 Core is the primary directly interacting domain.

### RPA70 directly interacts with HBoV1 NP1 through the RPA70-AB domain

**Fig 6:**
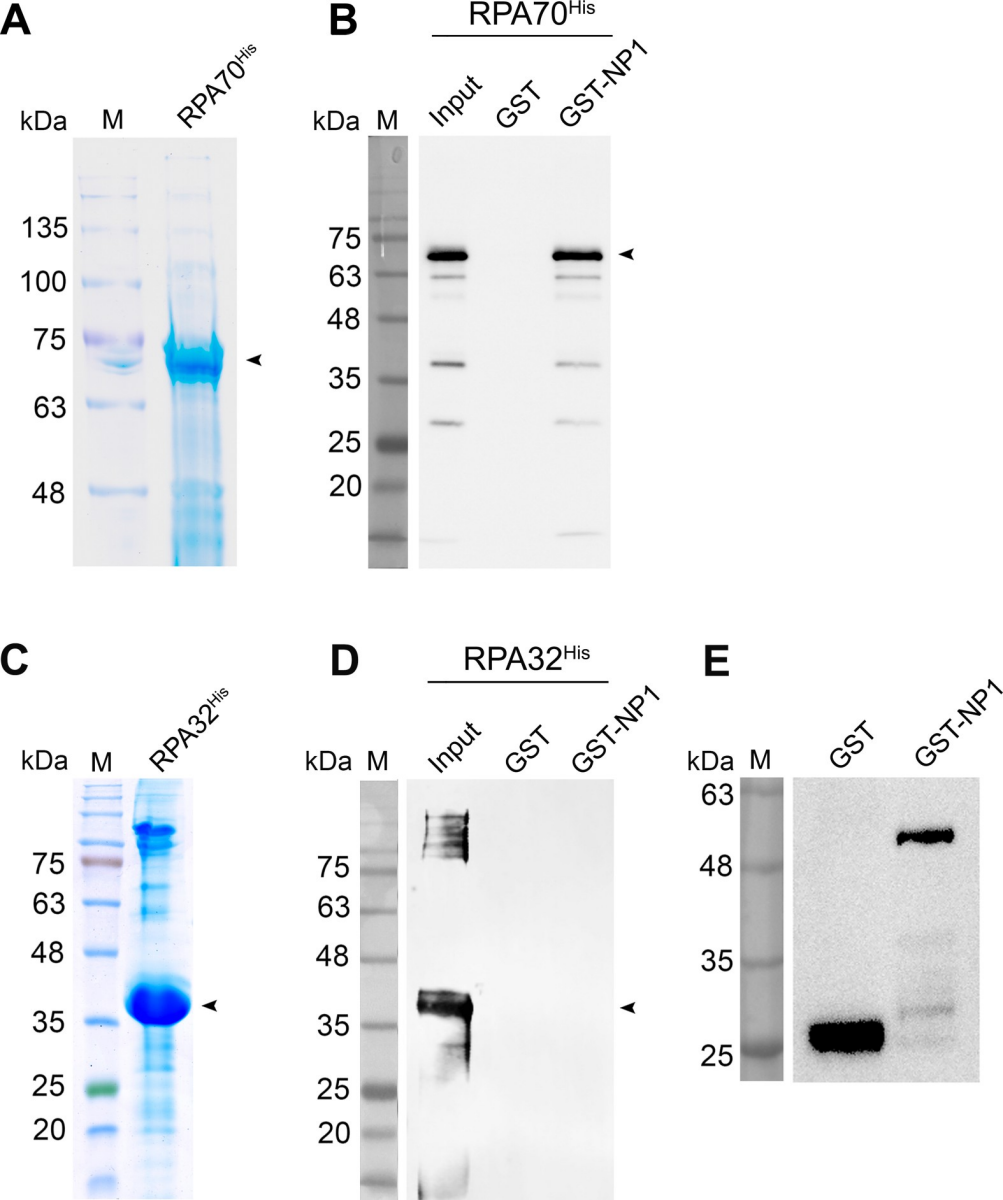
NP1 directly interacts with RPA70 of RPA heterotrimer. **(A&C) Purification of RPA70**^**His**^ **and RPA32**^**His**^ **proteins.** RPA70^His^ (A) and RPA32^His^ (C) were purified and separated on an SDS-PAGE gel followed by Coomassie brilliant blue staining. **(B&D) *In vitro* pull-down assay of RPA70**^**His**^ **and RPA32**^**His**^. 4 μg of purified GST-NP1 were used as bait to pull down 4 μg of prey proteins, purified RPA70^His^ (B) and RPA32^His^ (D), respectively, using Glutathione agarose. Pulldown proteins were analyzed by Western blotting with an anti-His antibody (B&D) or with anti-GST as a control (E). 4 μg GST of prey protein served as a negative control. Purified proteins and detected protein are denoted with arrowheads.

The large parvoviral nonstructural protein NS1 or Rep78/68 has been shown to directly interact with the RPA complex [47,48]. To investigate a direct interaction between NP1 and the RPA component, we aimed at RPA70 and RPA32, both harboring a potential protein-binding domain [50]. We firstly purified RPA70^His^ and RPA32^His^ (**Fig 6A and 6C**) and used them for an *in vitro* pull-down assay with GST-NP1. The results showed RPA70^His^, but not RPA32^His^, directly interacted with GST-NP1 (**Fig 6B and 6D**, GST-NP1); whereas as controls, neither of them interacted with GST (**Fig 6B and 6D,** GST), and the glutathione-agarose beads pulled down GST proteins (**Fig 6E**).

**Fig 7:**
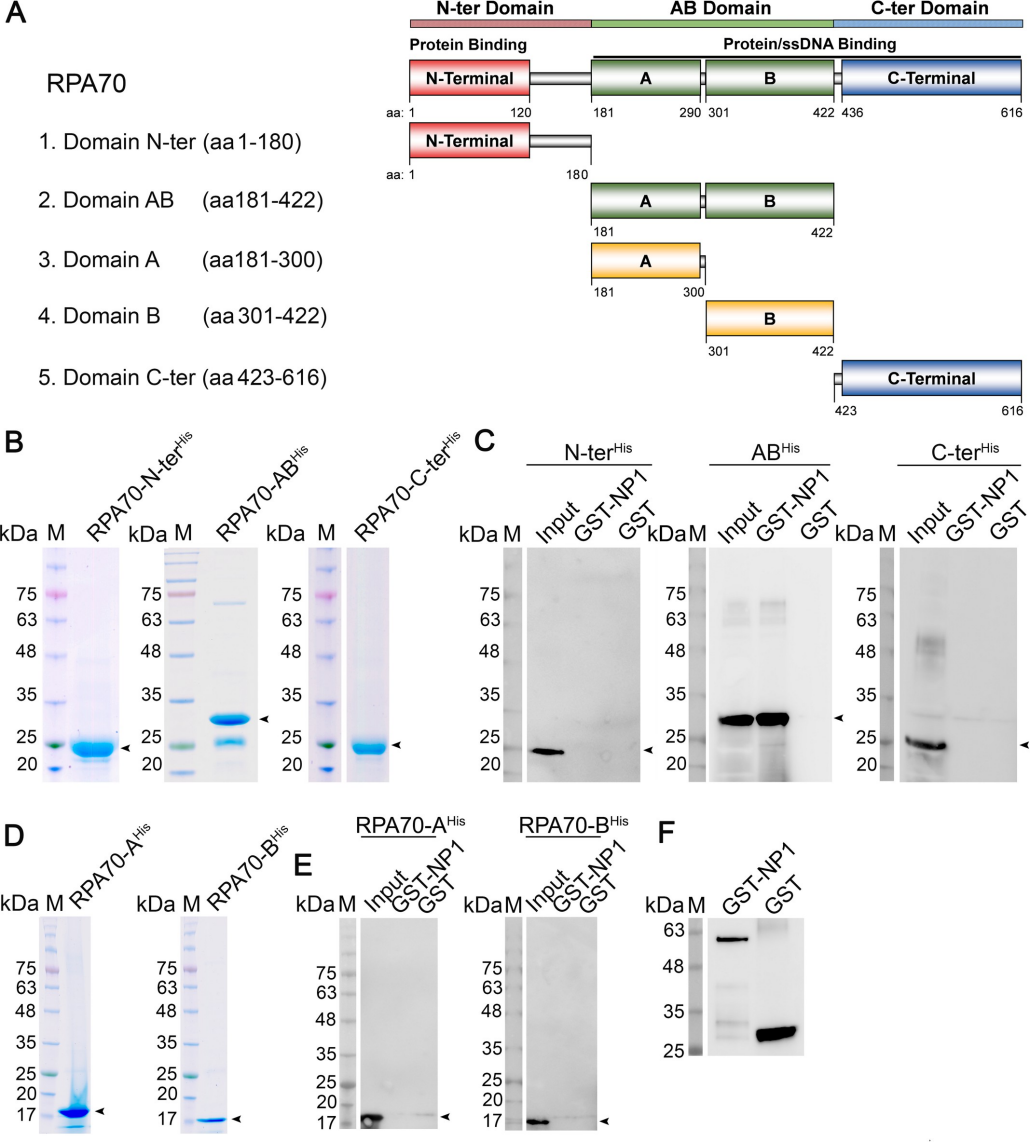
RPA70-AB domain directly interacts with HBoV1. **(A) A diagram of RPA70 domains.** The RPA70 N-terminal domain (RPA70-N-ter), AB domain (RPA70-AB), subdomains A and B within AB domain, C-terminal domain (RPA70-C-ter), and Linkers are diagrammed and shown in different colors. AB domin and C-terminus have both protein and ssDNA binding capability, while the C-terminus mediates heterodimerization with RPA32. (**B&D) Protein purification.** All purified truncated domains of RPA70, RPA70-N-ter, RPA70-AB, RPA70-C-ter, RPA70-A, and RPA70-B domains, indicated by arrowheads, were analyzed on SDS-(4–20%)PAGE gel. (**C, E&F**) ***In vitro* pull-down assays**. 4 μg of purified GST-NP1 or GST were used to pull down 4 μg of purified domains, as indicated, using Glutathione agarose. Pulldown proteins were analyzed by Western blotting with an anti-His antibody (C&E) or with anti-GST as a control (F). Arrowheads denote the interacting domains.

We further asked which RPA70 domain is the key NP1-binding domain. We purified three truncated domains of RPA70, the N terminal domain (RPA70-N-ter, aa1-180), the middle AB domain (RPA70-AB, aa181-422), and the C terminal domain (C-ter domain, aa423-616) (**Fig 7A and 7B**). Using the purified domains respectively in the pull-down assays with GST-NP1, the results revealed that only RPA70-AB^His^ was pulled down by GST-NP1 but was not with GST (**Fig 7C**). When the RPA70-AB was further truncated into two halves at the center for two smaller domains, RPA70-A (aa181-300) and RPA70-B (aa301-422) (**Fig 7D**), they were not pulled down by GST-NP1 (**Fig 7E**), suggesting that the central region of the RPA70-AB domain is crucial to bind NP1. As a control, the glutathione-agarose beads pulled down GST proteins (**Fig 7F**).

**Fig 8:**
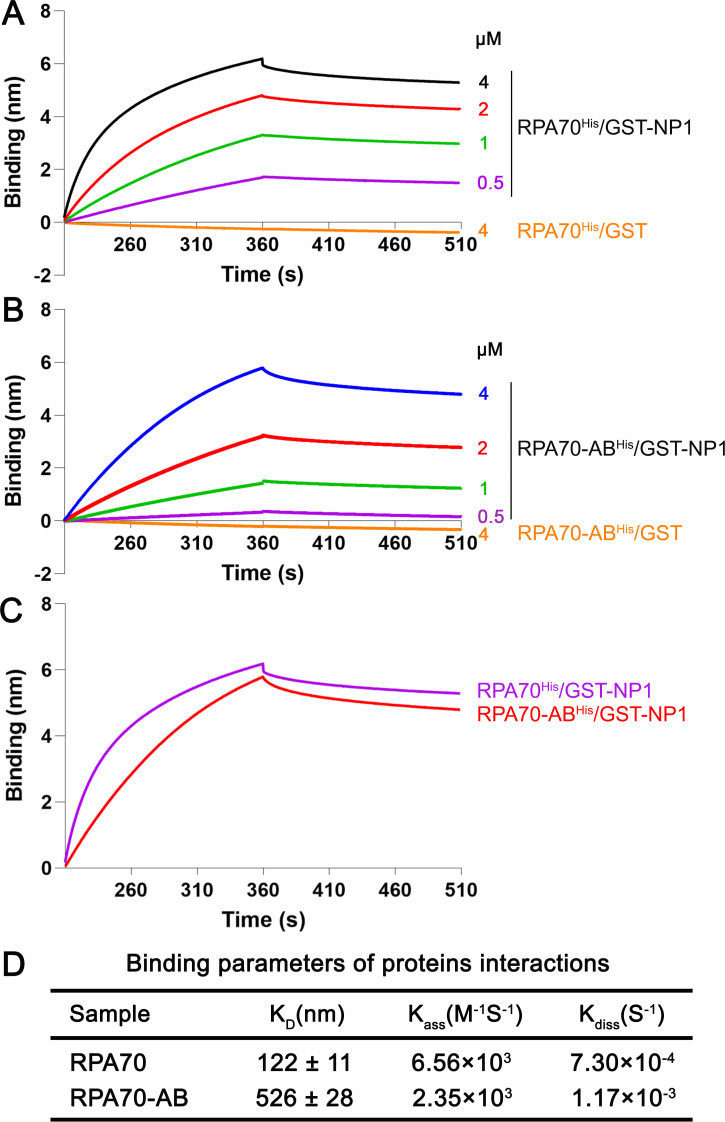
BLI analysis of the interaction between RPA70 and RPA70-AB with HBoV1 NP1. **(A-B) Kinetic assays using Ni-NTA biosensors.** The binding kinetics of associations and dissociations of 4 μM GST-NP1 with RPA70^His^ (A) and RPA70-AB^His^ (B) at different indicated concentrations are shown, together with controls of 4 μM GST with 4 μM Ku70^His^ or MBP-Ku70-β-barrel^His^. **(C) Comparison of the binding affinities.** The binding kinetics of associations and dissociations of of RPA70^His^ and RPA70-AB^His^ with GST-NP1 (all at 4 μM). **(D) Binding parameters of protein interactions.** Equilibrium dissociation constant K_D_ value represents the ratio of dissociation [K_diss_ (S^-1^)] and association [K_ass_ (M^-1^S^-1^)] computed from the real-time binding curves of GST-NP1 to RPA70^His^ or RPA70-AB^His^. The K_D_ values are shown with means ± standard deviations based on at least three repeated experiments.

Based on these results, we chose RPA70 and RPA70-AB for comparison of their binding kinetics with NP1. Then BLI assay was performed at 0.5, 1, 2, and 4 μM, respectively, for RPA70^His^ or RPA70-AB^His^ with GST-NP1 at 4 μM. In this assay, both of RPA70^His^ and RPA70-AB^His^ showed increasingly specific binding with GST-NP1 when their concentrations were increased (**Fig 8A and 8B**). The K_D_ values of RPA70^His^ and RPA70-AB^His^ with GST-NP1 were determined to be 122 ± 11 nM and 526 ± 28 nM (mean ± SD), respectively (**Fig 8D**). Echoing this result, 4 μM RPA70^His^ showed a higher binding affinity with 4 μM GST-NP1 versus 4 μM RPA70-AB^His^ as compared in a single BLI assay (**Fig 8C**). As controls, GST showed no binding with RPA70 and RPA70-AB (**Fig 8A and 8B**).

Taken together, we demonstrated that RPA70, but not RPA32, of the RPA complex directly interacts with GST-NP1 and that the RPA70-AB domain is the key interacting domain with NP1.

### NP1 enhances HBoV1 DNA replication *in vitro*

**Fig 9:**
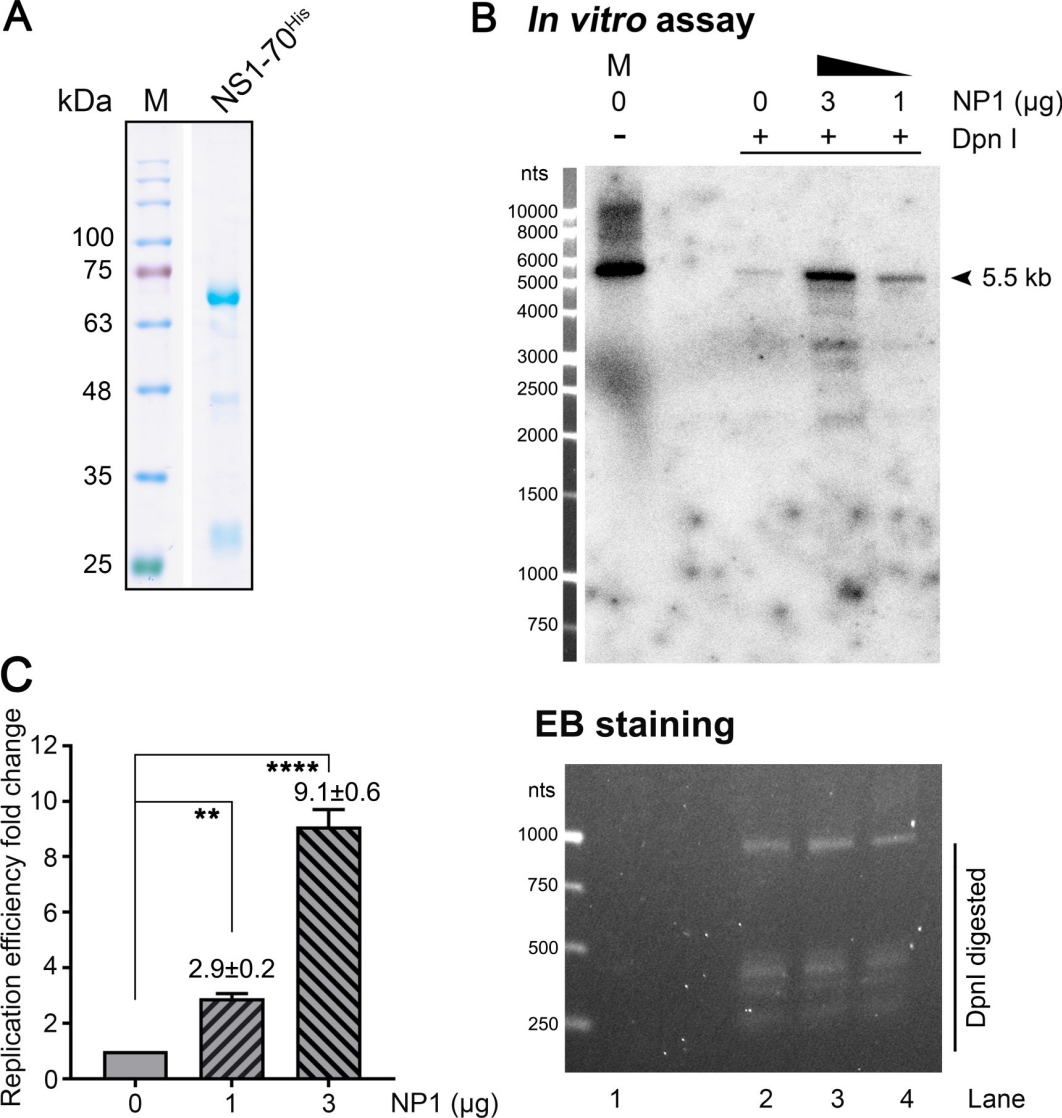
HBoV1 NP1 boosts HBoV1 DNA replication *in vitro*. **(A) Purification of HBoV1 NS1-70**^**His**^ **protein.** Purified HBoV1 NS1-70^His^ was analyzed on SDS-(12%)PAGE followed by Coomassie blue staining. **(B) *In vitro* replication assay.** NP1^His^ protein was added in two reactions at 1 μg and 3 μg, respectively, and the mock (0 μg) NP1 reaction served as the control. After DpnI digestion, the *in vitro* replicated products (lanes 2–4) were resolved on 1% agarose gel in TAE buffer. Ethidium bromide (EB)-staining visualized the DpnI digested input template (lower panel). The gel was then dehydrated to dry and exposed to a phosphor screen. The signals were developed on a Typhoon FLA 9000 scanner (upper panel). Lane 1: the reaction without DpnI digestion was loaded as a size maker (M) at 5.5 kb. The arrowhead indicates the *in vitro* replicated HBoV1 DNA. **(C) Quantification of the relative replication efficiency.** The DpnI-digestion resistant (replicated) viral DNAs (signals) were quantified using ImageQuant Tl (IQTL) 8.2 software (Cytiva). The quantities obtained from three repeats are presented as relative levels to the control (lane 2, panel B), and were obtained from triple experiments. **, P<0.01; ****, P<0.0001.

*In vitro* DNA replication assay is a powerful tool to dissect the orchestrated functions of both cellular and viral factors involved in viral DNA replication [51–55]. Here we, for the first time, established an *in vitro* HBoV1 DNA replication assay. We prepared cytosolic (S100) and nuclear (S300) extracts from HEK293 cells, and purified HBoV1 NS1-70^His^ (**Fig 9A**). Then, we used them to replicate the duplex HBoV1 genome, which was excised from pIHBoV1, in the presence of ^32^p-dCTP. After incubation at 37°C for ~16 h, DpnI digestion was used to degrade the input duplex genome (methylated) to small fragments (**Fig 9B**, EB staining/lanes 2–4). The DpnI-digestion resisted DNA bands shown at ~5.5 kb, which indicated the expected product from the viral DNA replication *in vitro* (**Fig 9B**, *In vitro* assay/lane 2–4). Strikingly, when purified NP1 was added at 1 and 3 μg, the DNA replication efficiency, as revealed on the *in vitro* replication assay, was augmented by ~3 fold and ~9 fold, respectively (**Fig 9B**, *In vitro* assay/lanes 3&4, and **Fig 9C**), compared with the NP1 absent reaction (**Fig 9B**, *In vitro* assay/lane 2). This result substantiated the direct role of HBoV1 NP1 in viral DNA replication.

Taken together, we, for the first time, established an HBoV1 *in vitro* DNA replication assay and proved that NP1 significantly augments HBoV1 DNA replication.

### Ku70-β-barrel and RPA70-AB domains inhibit HBoV1 DNA replication *in vitro*

**Fig 10:**
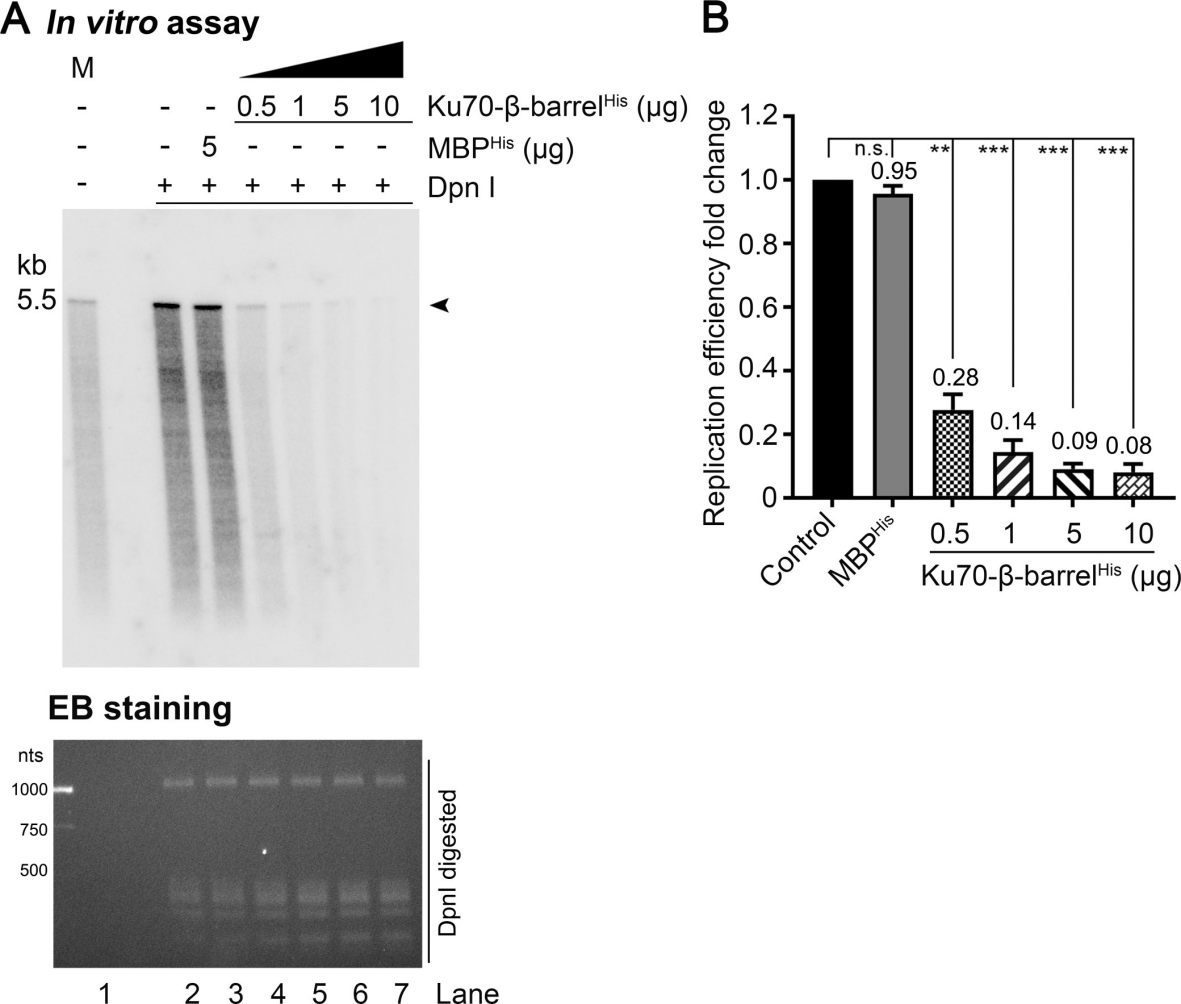
Complement of Ku70-β-barrel inhibits HBoV1 *in vitro* replication. (A) HBoV1 *in vitro* replication assay. The *in vitro* replication assays were carried out in the presence of 3 μg NP1 with complement of Ku70-β-barrel^His^ at 0.5, 1, 5, and 10 μg, respectively. Addition of 5 μg MBP^His^ protein served as a negative control. Products (lane 2–7) were treated with DpnI before separation on 1% agarose gel in TAE buffer. The gel was dried and exposed to a phosphor screen before scanning on a Typhoon FLA 9000 scanner. Arrowhead indicates the DpnI digestion resistant HBoV1 replicated DNA. Lane 1: the reaction without DpnI digestion was loaded as a marker (M). The EB stained gel at the bottom shows Dpn I digested DNA bands. **(B) Quantification of the relative efficiency of HBoV1 *in vitro* replication.** The DpnI-digestion resistant DNA bands were quantified using ImageQuant Tl (IQTL) 8.2 (Cytiva), and the quantities obtained from three repeats are presented as relative levels to the control (lane 3, pane A). **, P<0.01; ***, P<0.001.

**Fig 11:**
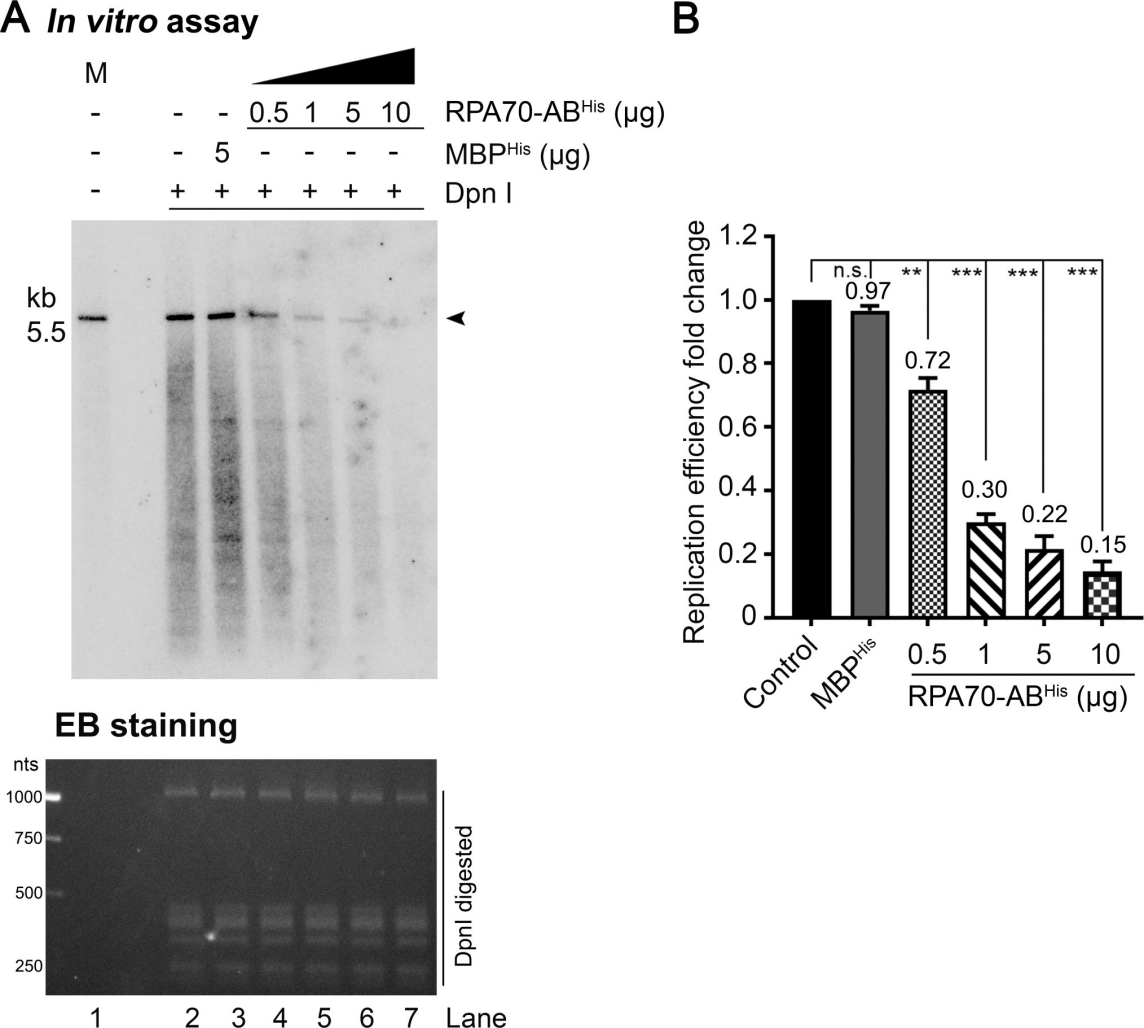
Complement of RPA70-AB reduces HBoV1 *in vitro* replication. RPA70-AB^His^ was added to the *in vitro* replication assays (with NP1) at 0.5, 1, 5, and 10 μg, respectively, and addition of 5 μg MBP^His^ served as a control. The analyzed gel of *in vitro* replication assays was imaged on a Typhoon FLA 9000 scanner (upper panel) and stained with EB (lower panel) (A) and quantified using ImageQuant Tl (B) as described in **Fig 10**. Lane 1, the reaction without DpnI digestion was loaded as a marker (M). Arrowheads indicate the DpnI digestion resistant HBoV1 replicated DNA. **, P<0.01; ***, P<0.001.

With the established HBoV1 *in vitro* replication assay, the functional interaction of the NP1 and Ku70 and RPA70 was verified. We supplemented the NP1-interacting domains, Ku70-β-barrel^His^ and RPA70-AB^His^, respectively, in the *in vitro* replication assay that had the NP1 (**Figs 10 and 11**). The addition of 0.5, 1, 5, and 10 μg of Ku70-β-barrel^His^ gradually inhibited HBoV1 DNA replication to efficiencies of 28%, 14%, 9% and 8%, respectively, compared with the mock control (**Fig 10**). Similarly, for RPA70-AB^His^, supplementation at 0.5, 1, 5, and 10 μg, respectively, decreased HBoV1 DNA replication to efficiencies of 72%, 30%, 22% and 15%, compared with the mock control **(Fig 11**). As a control, the addition of 5 μg MBP showed negligible effects on viral DNA replication, which remained at an efficiency of 95% and 97%, respectively, in the two supplementation assays (**Figs 10 and 11**, *In vitro* assay/lane 3).

Overall, with the supplementation of increased Ku70-β-barrel^His^ and RPA70-AB^His^ in the *in vitro* reaction system, HBoV1 DNA replication was decreased gradually. We concluded that the NP1 interacting domains, the β-barrel domain of Ku70 and the AB domain of RPA70, competitively inhibited HBoV1 DNA replication *in vitro*. Thus, the *in vitro* replication assay confirmed that the HBoV1 NP1 interacts with the cellular factors of Ku70 at the β-barrel domain and of RPA70 at the AB domain in the process of viral DNA replication.

### Ku70-β-barrel and RPA70-AB domains inhibit HBoV1 replication in infected HAE-ALI

**Fig 12:**
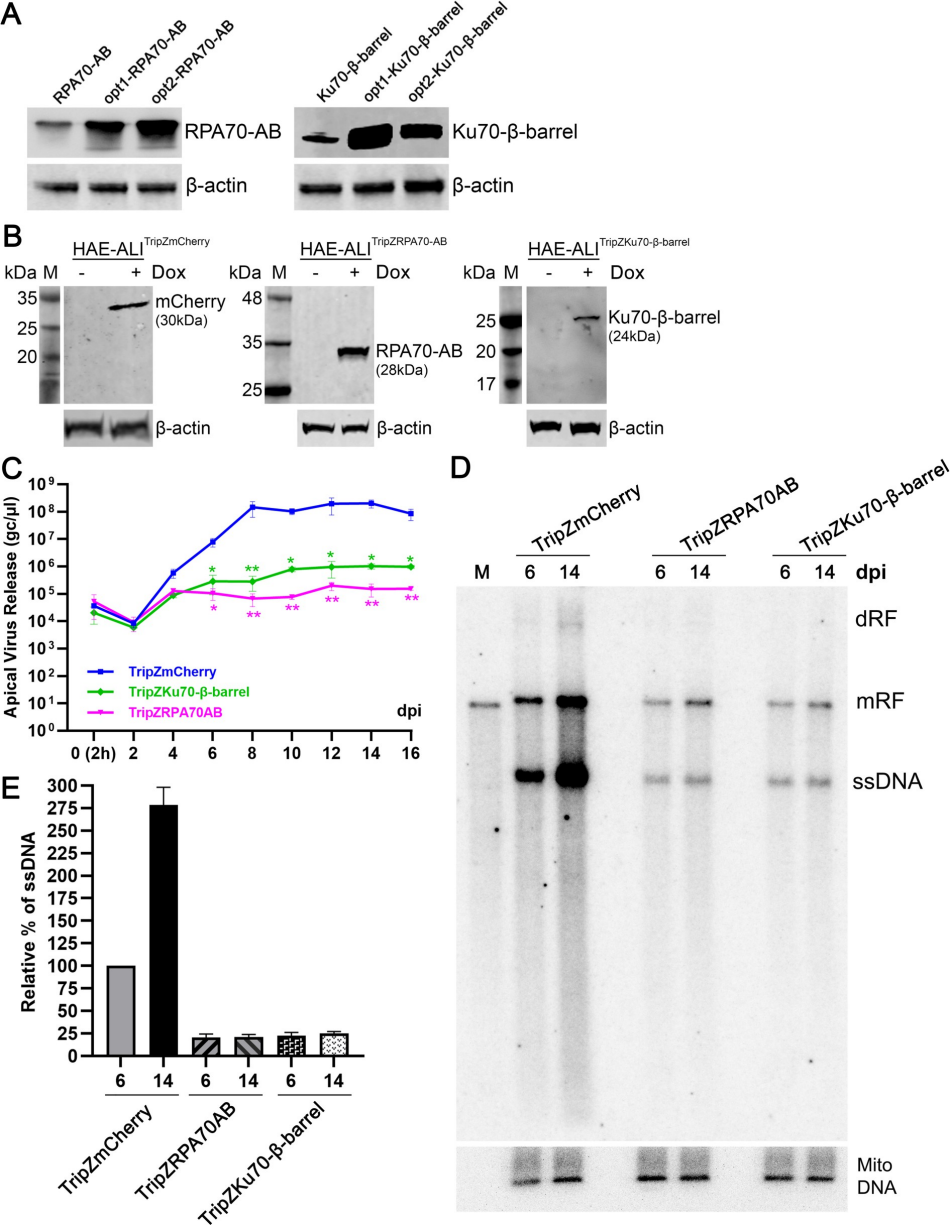
Overexpressions of the AB domain of RPA70 and the β-barrel domain of Ku70 significantly decrease HBoV1 replication in HAE-ALI. **(A) Codon optimization of RPA70-AB and Ku70-β-barrel.** HEK293 cells were transfected with plasmids expressing codons optimized RPA70-AB^Strep-HA^ and Ku70-β-barrel^Strep-HA^ ORFs, respectively. At 2 days post-transfection, the cells were collected and lysed for Western blotting using anti-Strep. β-actin was probed as a loading control. **(B) Induction of expression of mCherry, RPA70-AB, and Ku70-β-barrel domains in HAE-ALI.** HAE-ALI cultures were derived from the airway epithelial cells transduced with lentivirus TripZmCherry, TripZRPA70-AB, and TripZKu70-β-barrel. At one day prior to HBoV1 infection, doxycycline (Dox) was added to the basolateral chamber of the ALI cultures. At 2 days post-treatment, the cells were probed for expression of mCherry, RPA70-AB, and Ku70-β-barrel by Western blotting using anti-Strep. β-actin was reprobed as loading controls. **(C-E) Overexpression of RPA70-AB, and Ku70-β-barrel domains in HAE-ALI decrease the HBoV1 apical virus release and viral genome replication.** Indicated domain expressing HAE-ALI cultures were infected with HBoV1 at an MOI of 100 DRP/cell. Dox was added prior to the infection and refreshed every 3–4 days. **(C) HBoV1 apical release** Apical released HBoV1 were collected from the ALI cultures over a course of 16 days every other day. At the indicated days post-infection (dpi), the apical released viruses were collected in 300 μl of D-PBS and quantified for DRP using qPCR. Values represents means ± standard deviations of the viruses collected from 3 infected ALI cultures. Relative values are shown with means and standard deviations. *, p<0.05; **, p<0.01. **(D) Southern blotting.** At 6 and 14 dpi, Hirt DNA were extracted from HBoV1-infected HAE-ALI cultures that were differentiated from the primary airway epithelial cells transduced with various lentiviral vectors as indicated, followed by Southern blotting. The blot was probed with a HBoV1 DNA probe and mitochondrial DNA probe (Mito-DNA), respectively. dRF, mRF and ssDNA represent double, monomer replicative from and single stranded DNA, respectively. Duplex HBoV1 genome was loaded as a size marker (M) of 5.5 kb. **(E) Quantification of the signals from autoradiography**. The intensity of ssDNA bands on the blot was quantified using ImageQuant Tl software, and Mito-DNA served as a loading control. The values (mean ± SD) obtained from three blots were normalized to the replication of viral ssDNA in TripZmCherry expressing (HBoV1-infected) HAE-ALI at 6 dpi, which is arbitrarily set up to 100%.

As both Ku70-β-barrel and RPA70-AB interacted with HBoV1 NP1 and inhibited viral DNA replication *in vitro*, we hypothesized that overexpression of either the Ku70-β-barrel or the RPA70-AB in HAE-ALI inhibited HBoV1 replication as well. To prove this, we first executed codon optimizations of the ORFs of the Ku70-β-barrel and the RPA70-AB for efficient expression in cells. The Ku70 β-barrel and RPA70-AB domains are intact structural domains that have been well defined [38,50,56]. We confirmed that the opt1-Ku70-β-barrel and the opt2-RPA70-AB abundantly expressed in transfected HEK293 cells (**Fig 12A)**. Next, we integrated the Tet-on inducible expression of opt2-RPA70-AB or opt1-Ku70-β-barrel into proliferating primary human airway epithelial cells using lentiviral vector transduction, so that the expression of these domains could be kept silent during the epithelium differentiation to avoid the potential negative effects. The two domains and a control *mCherry* ORF were individually cloned into the inducible lentiviral transfer vector (pTripZ) for the production of lentiviral vectors. The lentivirus-transduced airway epithelial cells were differentiated at an ALI for 4 weeks to obtain polarized HAE-ALI cultures. At one day prior to HBoV1 infection, expression of RPA70-AB, Ku70-β-barrel, and mCherry in HAE-ALI cultures was induced by the addition of 2 μg/ml doxycycline (Dox) in the basolateral medium (**Fig 12B**). During the of 16-day course of infection, apical washes were collected for quantification of virus release using qPCR at an interval of every 2 days, and Hirt DNA were sampled at 6 and 14 dpi, respectively, for Southern blotting. The results demonstrated that more apical release of virus progeny was detected from the HAE-ALI expressing the mCherry control at the early phase of infection, and starting from 6 dpi, the Ku70-β-barrel, RPA70-AB expressing HAE-ALI decreased apical virus release at 2–3 logs less than that from the mCherry-expressing control (**Fig 12C**). At 6 and 14 dpi, the levels of viral ssDNA in the Ku70-β-barrel and RPA70-AB expressing HAE-ALI were detected at least > 4 times less than that in the mCherry expressing control (**Fig 12D and 12E**).

Taken together, using a dominant negative strategy, we confirmed that overexpression of the RPA70-AB and Ku70-β-barrel domains in HAE-ALI inhibited HBoV1 DNA replication.

### NP1 mutants that do not interact with RPA70 and Ku70 *in vitro* lose their function in facilitating HBoV1 DNA replication *in vivo*

**Fig 13:**
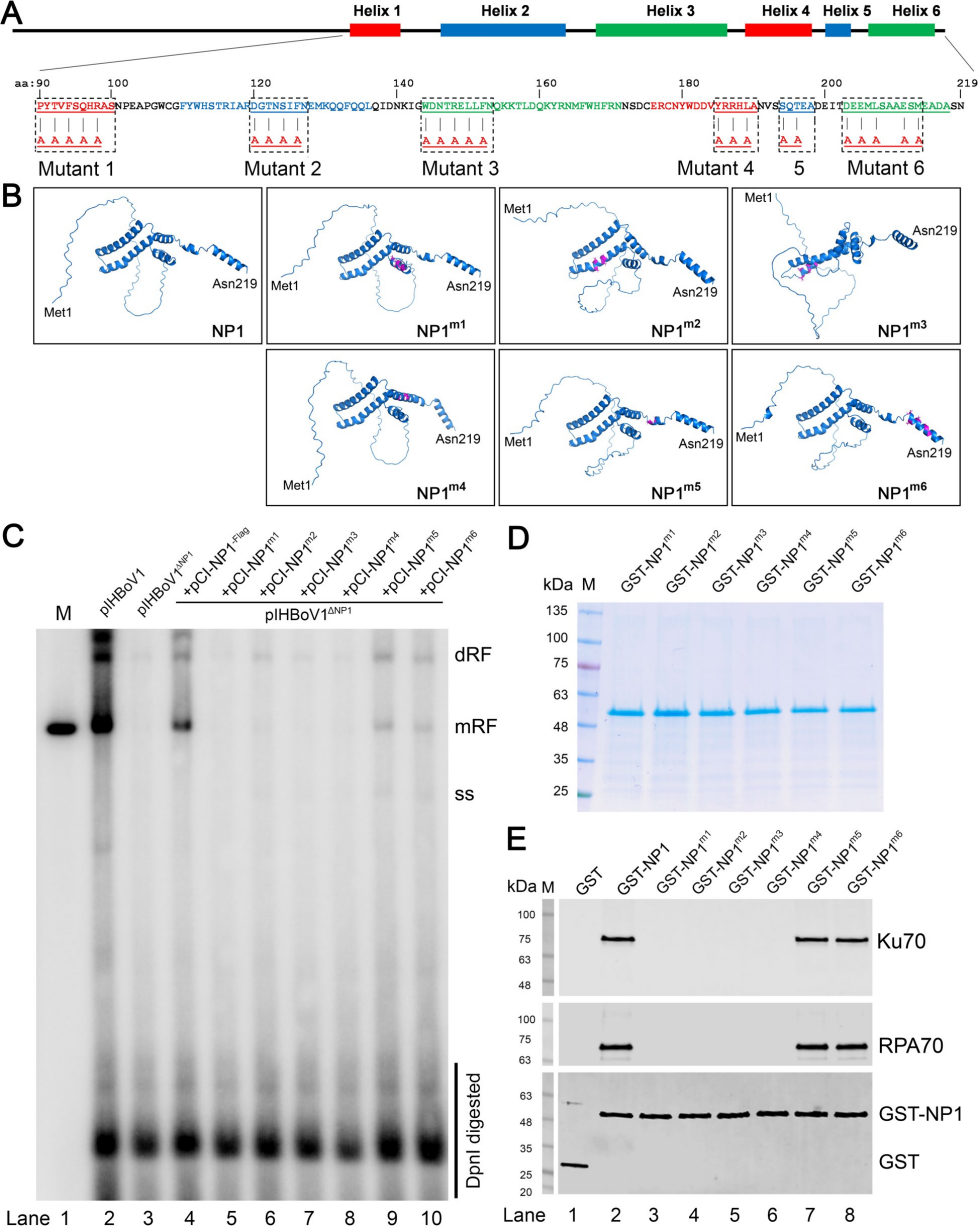
NP1 mutants that do not interact with Ku70 and RPA70 *in vitro* lose the function in facilitating HBoV1 DNA replication in HEK293 cells. **(A) Schematic digram of the NP1 protein.** NP1 protein is schematically diagramed with predicated helical strutrues. The amino acid sequences of aa90-219 are shown with indication of each helix. The potential functional motifs (underlined) within each helical struture were predicted with Eukaryotic Linear Motif resource. NP1 mutants that bear mutaions in helix structure 1–6 are indicated. **(B) Prediction of NP1 structures**. The structures of NP1 and its mutants were predicated with AlphaFolder2. **(C) Southern blotting.** HEK293 cells were transfected with pIHBoV1, pIHBoV1^ΔNP1^, and co-transfected with pIHBoV1^ΔNP1^ and pCI-NP1^Flag^ or pIHBoV1^ΔNP1^ and each indicated pCI-NP1 mutants, respectively. At 2 days post-transfection, Hirt DNA was extracted from the cells of each transfection and analyzed by Southern blotting. A full-length HBoV1 genome was used as probe and HBoV1 duplex DNA excised from pIHBoV1 was used as a size marker (M) of ~5.5 kb (Lane 1). dRF, mRF, and ssDNA represent double, monomer replicative form DNA, and single stranded DNA, respectively. DpnI digested DNA is indicated. **(D) Purification of GST-NP1 and its mutants.** HBoV1 GST-NP1 and GST protein were purified and anlyzed on Comassite brilliant blue stained SDS-4-20%PAGE gel. **(E) *In vitro* pull-down assay of GST-NP1 and its mutants with Ku70 and RPA70.** 4 μg of purified GST-NP1 and its mutants were used as bait to pull down 4 μg of the prey protein, purified Ku70^His^ and RPA70^His^, respectively, with Glutathione agarose. 4 μg of GST served as a negative control. Proteins pulled down were separated on SDS-PAGE gel and blotted with anti-His antibody for Ku70^His^ and RPA70^His^ and anti-GST for GST-NP1 and its mutants.

We next attempted to identify the key motifs of the NP1 that interacted with Ku70 and RPA70. Since the structure of the NP1 of any member in the genus *Bocaparvovirus* has not been resolved, we used the Artificial intelligence (AI) based protein prediction program AlphaFolder2 (https://alphafold.ebi.ac.uk/) [57,58] to predict the NP1 structure. AlphaFolder2 has been widely used for protein structure prediction [59,60]. The program showed 6 helix structured domains localized between aa90-218 of the NP1 (**Fig 13A and 13B**, NP1). To precisely localize the potential functional motifs within these helixes, an online tool Eukaryotic Linear Motif resource (ELM; http://elm.eu.org/) was used for the prediction [61]. Analyses revealed several motifs within each helix (**Fig 13A**), guilding us to mutate selected amino acids insides these motifs to alanines for six NP1 mutants. The predicted structure of these mutants harboring respective mutations in each helix are presented in **Fig 13B**. We then used the NP1 mutants (pCI-NP1^m1-6^), as well as the wild-type pCI-NP1 (as a control), to complement the lack of the NP1 in pIHBoV1^ΔNP1^-transfected HEK293 cells, followed by Southern blotting for viral DNA replication (**Fig 13C**). The results showed that the NP1 mutants bearing mutations in helix 1, 2, 3 and 4 regions, respectively, nearly abolished viral DNA replication as shown with undetectable bands of the monomer replicative form (mRF) DNA bands; however, Mutant 5&6, where the muttaions located at the C-terminal helixes 5&6, remained obvious mRF bands (**Fig 13C**, mRF).

We then purified all mutated NP1 proteins and examined for their binding with Ku70 or RPA70 in an *in vitro* pulldown assay (**Fig 13D and 13E**). The results showed that neither Ku70 nor RPA70 was pulled down by the GST-fused NP1 Mutants 1–4; however Mutants 5&6 remained their interaction with Ku70 or RPA70 (**Fig 13E**, lanes 3–6 vs 7&8).

Taken together, the NP1 mutants (Mutants 1–4) that lost the *in vitro* binding with Ku70 and RPA70 remarkedly reduced their capability to support HBoV1 DNA replication *in vivo*, but not the two mutants (Mutants 5&6) that remained their interactions with Ku70 and RPA70. This result strongly supported that the interaction of NP1 with Ku70 and RPA70 is critical for HBoV1 DNA replication.

### NP1 performs as a mediator to recruit Ku and RPA complexes during HBoV1 replication

**Fig 14:**
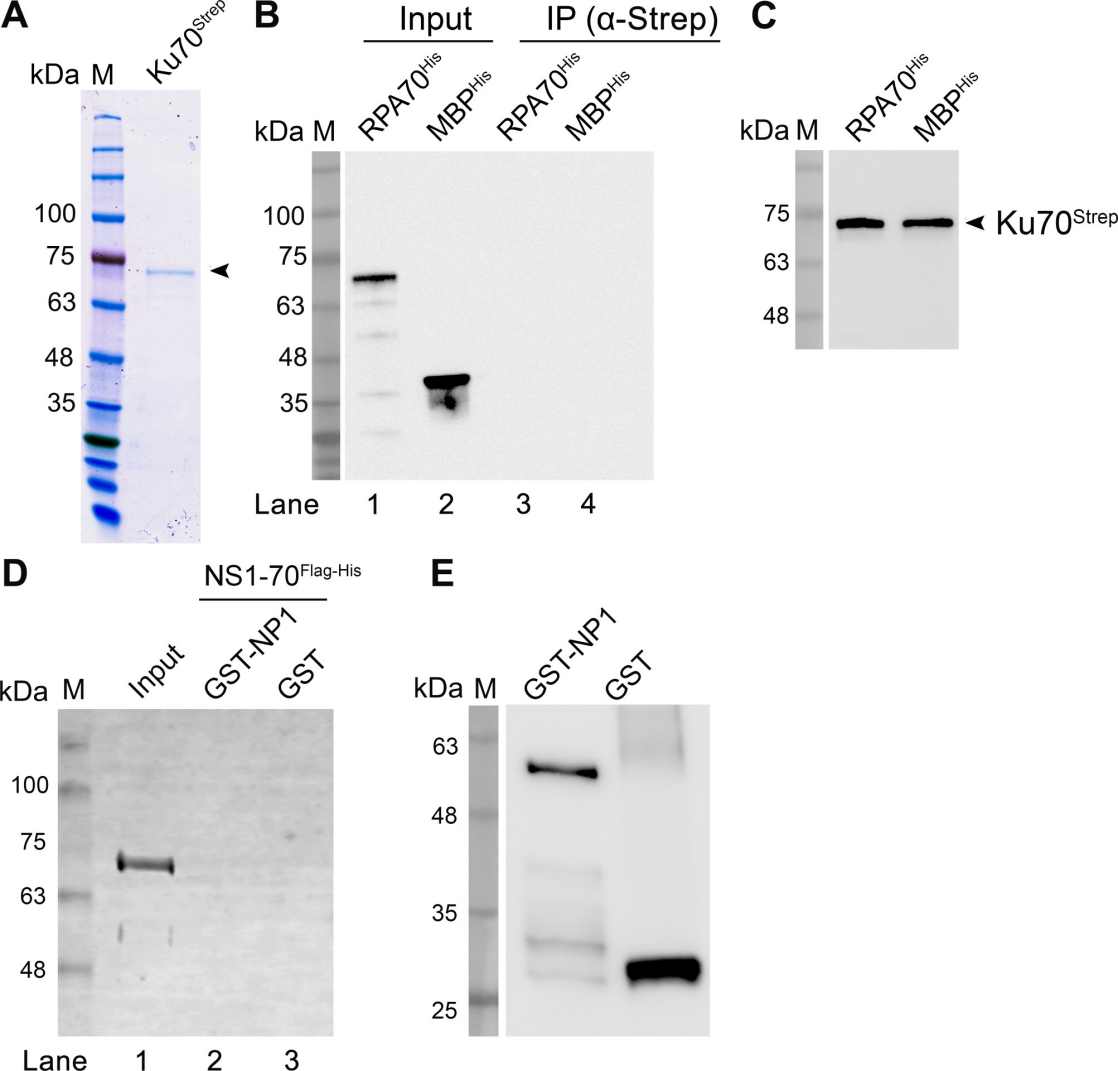
Ku70 does not interact with RPA70, and NS1 does not interact with NP1 *in vitro*. **(A) Purification of Ku70**^**Strep**^. Purified strep tagged Ku70 protein was separated on SDS-(4–20%)PAGE gel, followed by Coomassie blue staining. **(B&C) *In vitro* pull-down of Ku70 with RPA70.** 4 μg of purified Ku70^Strep^ was used as bait to pull down 4 μg of purified RPA70^His^ using Strep-Tactin agarose. MBP^His^ served as a negative control. The pull-down proteins were analyzed by Western blotting using anti-His for RPA70^His^ and MBP^His^ (B) and anti-Strep for Ku70^Strep^ (C). **(D&E) *In vitro* pull-down of NS1 with NP1.** 4 μg of purified GST-NP1 was used as bait to pull down 4 μg of purified HBoV1 NS1-70^FlagHis^ using Glutathione agarose. 4 μg of GST prey protein served as a negative control. The pulldown protein was analyzed by Western blotting using anti-His (D) and anti-GST (E).

**Fig 15:**
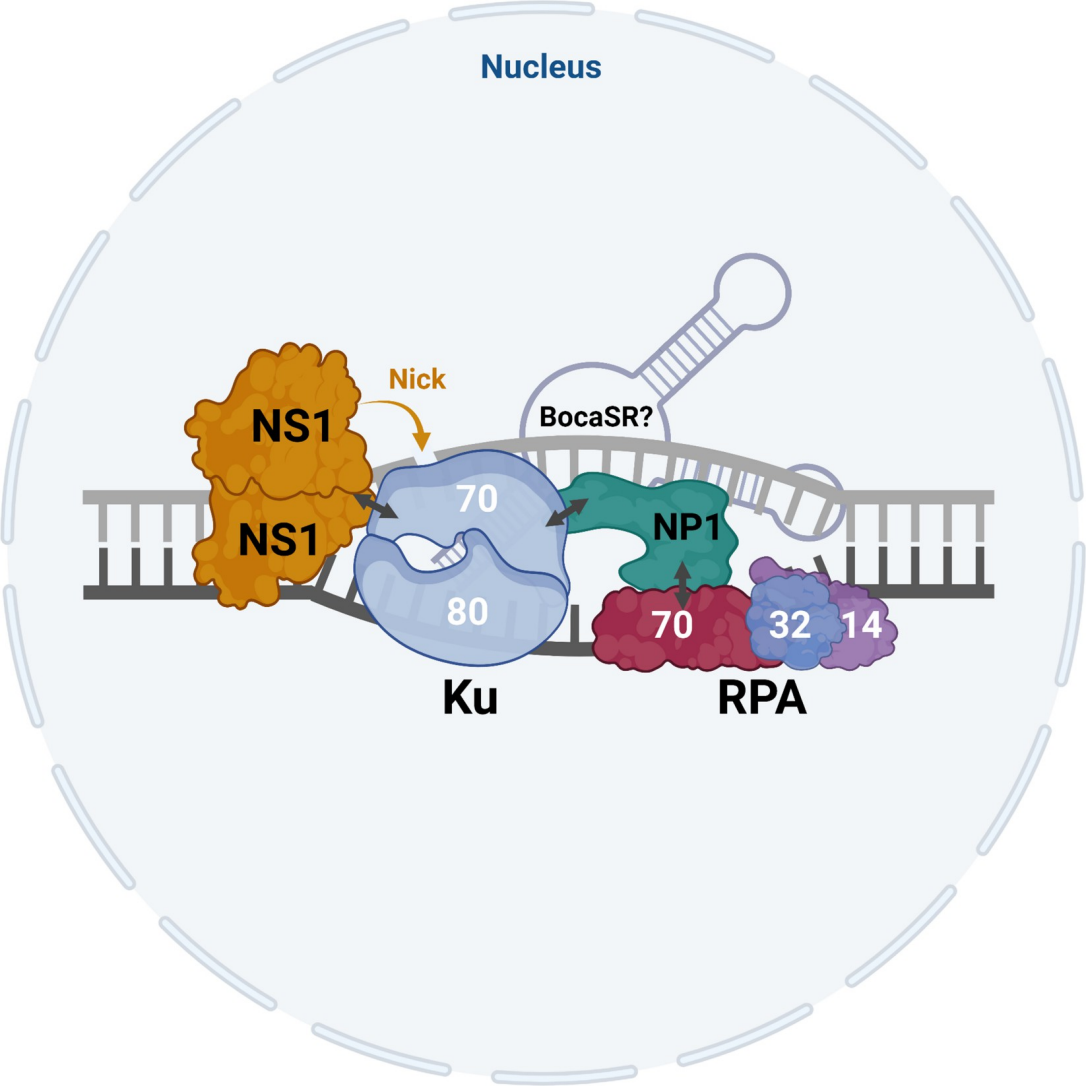
Proposed model of the HBoV1 replication initiation complex. The replication origin of HBoV1 is depicted as double helix DNA. NS1 binds to the NS1-binding domain as a multimer to one binding site in the replication origin, as diagrammed. Ku70, complexed with Ku80, interacts with both NS1 and NP1, which is shown by double arrows. Additionally, NP1 interacts with RPA70, which is complexed with RPA32 and RPA14 as diagrammed. The RPA complex binds to ssDNA, which is likely unwound by the helicase activity of the NS1 and Ku complex. HBoV1 BocaSR, diagrammed behinds Ku and RPA complexes, may tighten all these interactions (complexes) or may recruit additional repair/replication factors, which play an indispensable role in the HBoV1 replication initiation machinery. Double arrows indicate protein-protein interactions. The schematic model was created with BioRender.

Since both Ku70 and RPA70 directly interacted with NP1 during HBoV1 replication, we then asked if Ku70 and RPA70 interacted directly with each other. To this end, we performed an *in vitro* pull-down assay of Ku70 with RPA70 using the purified Ku70^strep^ and RPA70^His^ (**Fig 14A**), and the results showed Ku70^strep^ did not pull-down RPA70^His^ (**Fig 14B**, lane 3). As a control, Strep-Tactin agarose pull down Ku70^Strep^ (**Fig 14C**). In addition, we also found that GST-NP1 did not pull down NS1-70^Flag-His^ (**Fig 14D**, lane 2). These results suggest that NP1 acts as a mediator to recruit the replication-necessary proteins, i.e., Ku70 and RPA70, during HBoV1 replication (**Fig 15**). At least during this course, Ku70 and RPA70 directly interacted with the mediator NP1 protein, which were accumulated in the proximity of NS1 and the viral replication origin, but NP1 and NS1 did not interact with each other.

## Discussion

In the present study, we identified that a small parvoviral nonstructural protein of 25 kDa, HBoV1 NP1, directly interacts with the Ku70 and RPA70 proteins of the Ku and RPA complexes, respectively. Addition of the respective NP1-interacting domains of Ku70 or RPA70 significantly decreased HBoV1 DNA replication in an *in vitro* viral DNA replication assay using HEK293 cell extracts and purified NS1 and NP1. Moreover, overexpression of the NP1-interacting domains of Ku70 and RPA70 drastically suppressed the productive infection of HBoV1 in HAE-ALI cultures, indicated by the reductions in both viral DNA replication and apical virus release during a course of 16 dpi. As NS1 neither directly interacts with RPA nor NP1, our findings suggest that NP1 functions as a mediator to bridge the Ku and RPA complexes to a close proximity to the NS1, which facilitates HBoV1 replication. In this study, we also identified the interaction of NP1 with cellular proteins that are involved in mRNA processing, HNRNPU, PUF60, DDX30, YBX1, and DDX9. We have shown that HBoV1 NP1 regulates viral mRNA processing and is indispensable to viral capsid protein expression [16,23], thus we will study these mRNA processing related proteins in the future.

Parvoviruses, packaged with a compact ssDNA genome of ~4–6 kb, have utilized multiple strategies to replicate its genome that solely relies on cellular DNA replication or DNA repair machinery. Autonomously replicating (autonomous) parvoviruses, except for HBoV1, utilize cellular DNA replication factors that are enriched in the S or late S (G2/M) phase for viral DNA replication [62–67]. Parvovirus infection often triggers a DDR that facilitates viral DNA replication [25,27,28,66]. The DDR induces cell cycle arrest at the S or G2/M phase of the infected cells, which accumulates DNA replication factors, i.e., DNA polymerase δ, the replication factor C (RFC), proliferating cell nuclear antigen (PCNA), minichromosome maintenance protein complex (MCM), and RPA, for viral DNA replication [67–70]. HBoV1 is unique in that it replicates in differentiated (mitotically terminated) epithelial cells of the human airway epithelia [30,33,35]. Intriguingly, the HBoV1 duplex genome, when delivered by transfection, replicates in dividing HEK293 cells and generates progeny virus that are infectious to human airway epithelia; however, HBoV1 does not infect HEK293 cells [31,35,71]. The HBoV1 DNA replication in either differentiated or proliferating cells relies on the cellular DNA damage signaling and repair pathways, including phosphorylation of RPA32, DNA-PKcs activation and two Y-family DNA repair DNA polymerases, Pol η and Pol κ [30,31]. The replication of dependoparvovirus AAV is delegated to the coinfection with a “helper” virus, such as adenovirus (Ad) and herpes simplex virus (HSV) [72–74], in which helper viral DNA replication DNA polymerase is used in both proliferating and differentiated cells [64,75,76]. Notably, HBoV1 is also a helper virus for AAV. HBoV1 infection fully supports AAV2 replication in differentiated human airway epithelia [77], highlighting a similarity in viral DNA replication between AAV and HBoV1 in mitotically terminated cells, which should use DNA repair machinery for viral DNA replication. Thus, it suggests that the HBoV1 replication recruits the DNA repair machinery to help the AAV genome replication.

*In vitro* DNA replication assays have been extensively used for the study of essential elements involved in replication of parvovirus AAV and MVM [51–55,78]. Using *in vitro* replication assays, in addition to the large non-structural protein (Rep78/68 or NS1), RFC, MCM, PCNA, RPA, and Pol δ were found essential in reconstituting AAV2 DNA replication [52], and PCNA, RPA, Pol δ, and parvovirus initiation factor (PIF) were essential to MVM DNA replication *in vitro* [48,79]. We for the first time established the *in vitro* DNA replication assay to study HBoV1. While the NS1 is essential to replicate the HBoV1 duplex genome, we found that the small non-structural protein NP1 is necessary to observe obvious replication signals. We also found a mixture of cytosol (S100) and nuclear (S300) extract gave the best result. The assay established in this study is expected to be a robust tool to examine functions of other DNA repair factors in HBoV1 viral DNA replication in the near future.

As a large component in the heterotrimeric protein complex, RPA70’s N-terminal and AB domains function as DNA binding domains and its C-terminal domain is responsible for forming the trimerization core with the RPA32’s D domain [44,45,50]. During parvovirus infection, RPA32 is found to be highly phosphorylated [27,28,69]; however, the phosphorylated RPA32 is at least functional to support parvovirus B19 replication [70]. The RPA heterotrimer directly interacts with MVM NS1 [48] and AAV2 Rep78 [47]. While the MVM interaction with RPA is critical to the unwinding of the MVM duplex DNA replication origin and replication fork formation [48], the interaction of AAV Rep78 and RPA enhances the nicking (the endonuclease activity of Rep78) at the viral DNA replication origin [47]. Therefore, recruitment of the RPA complex by the Rep78 or NS1 to the viral DNA replication origin, where the Rep78/NS1 binds and executes endonuclease activity, is essential to AAV/MVM DNA replication. Notably, HBoV1 NS1 did not directly interact with RPA complex, which likely does not recruit RPA complex efficiently, although it does interact with RPA in a manner independent of nucleic acids in a co-immunoprecipitation assay [49]. In this regard, HBoV1 has employed NP1 to compensate for the lack of an NS1-RPA direct interaction, although NS1 and NP1 do not interact directly with each other. The facts that NS1 directly interacts with Ku70 [49] and that NP1 directly interacts with both Ku70 and RPA70 support a mechanism under which NP1 serves as a bridge to fulfill the recruitment of the RPA complex to the viral DNA replication origin through the NS1-Ku70-NP1 interaction.

Ku complex is a heterodimer of Ku70 and Ku80. In addition to its involvement in NHEJ DNA repair [80], the complex functions as a ssDNA-dependent ATP-dependent helicase [43]. The central seven-stranded anti-parallel β-barrel domains of both Ku70 and Ku80 form a basket-shaped structure that binds DNA, and the C-terminus forms a main helical carboxy-terminal arm region, which mediates Ku heterodimerization [80]. The Ku70/80 heterodimer was found in the AAV Rep78-associated complex and colocalized in the AAV replication centers [81]. In an *in vitro* DNA replication assay, Ku promotes strand displacement synthesis by acting as a replicative helicase in the absence of the MCM complex [52], and the depletion of Ku decreased AAV2 DNA replication [82]. Both the Ku and MCM complexes have similar 3’→ 5’ DNA helicase activities and both have a preference for replication fork substrates [43,83]. Considering MCM is a DNA replication origin bound DNA helicase, it unwinds the dsDNA at the DNA replication origin, recruits DNA polymerases and initiates DNA synthesis [84]. While a direct interaction of AAV Rep78 or MVM NS1 with Ku complex has not been explored, we recently identified that HBoV1 NS1 directly interacts with the Ku70 protein, with the highest binding affinity at the C terminal (aa437-609) domain with NS1 [49]. Thus, Ku70 directly interacts with NP1 and NS1 proteins through the core β-barrel (aa266-439) and mainly C-terminal (aa437-609) domains, respectively, which should ensure an efficient recruitment of the Ku complex to the viral DNA replication origin. The level of Ku70 and Ku80 expression in HAE-ALI cultures were not affected by HBoV1 infection (**S4 Fig**), which ensures a consistent interaction between NP1 and the Ku complex during the course of infection. The biasedly preferred NS1 and NP1 binding Ku70 domains solidate the requirement of the Ku complex, which is an ingenious strategy utilized by HBoV1 for its genome amplification.

Based on these lines of evidence, we proposed a model of the HBoV1 replication machinery (**Fig 15**), which involves HBoV1 NS1, NP1, and the cellular RPA and Ku complexes. Notably, we previously showed that the HBoV1 transcribed-small noncoding RNA (BocaSR) accumulates in the viral DNA replication centers and plays a role of augmenter in HBoV1 DNA replication [17]. The interactions between NP1 and BocaSR, or among Ku70, RPA70, and other cellular components with BocaSR require further investigation. In conclusion, our results explain the mechanism underlying how NP1 recruits Ku and RPA complexes into the HBoV1 replication initiation machinery. NP1 functions as a mediator to bridge DNA replication/repair proteins close to NS1 through the NP1-Ku70-NS1 interaction, which binds and nicks the viral DNA replication origin to initiate viral DNA synthesis.

## Materials and methods

### Cell lines and primary cultures

#### HEK293 cells

HEK293 cells (#CRL-1573, ATCC, Manassas, VA) and HEK293T cells (#CRL-11268, ATCC) obtained from ATCC were cultured in Dulbecco’s modified Eagle’s medium (DMEM) (#SH30022.01; Cytiva Life Science, Marlborough, MA) at 37°C with a 5% CO_2_ atmosphere. The culture media were supplemented with 10% fetal bovine serum (FBS; #F0926, MilliporeSigma, St. Louis, MO). HEK293-ES cells (#94-007S) were purchased from Expression Systems, LLC (Davis, CA) and cultured in ESF SFM medium (#98-001-01; Expression Systems) in vented shake flasks on a shaker at 140 rpm at 37°C and 5% CO_2_.

#### HAE-ALI culture

Primary human airway epithelium (HAE) cells were generated using published methods [30,34,35,85], and cultured in 12-mm Transwell inserts (#3460; Corning, NY) at an air-liquid interface (ALI), termed HAE-ALI. Briefly, primary bronchial airway epithelia cells, obtained from the Cell Culture Core of the Center for Gene Therapy, University of Iowa, were propagated in airway cell expansion medium (PneumaCult-Ex Plus media, #05040, StemCell, Vancouver, BC, Canada) on collagen IV (# C7521, MilliporeSigma, St. Louis, MO)-coated 100 mm dishes in 5% CO_2_ at 37°C in less than three passages. The confluent cells were dissociated and seeded onto Transwell inserts at 4.5 × 10^5^ cells/insert. Both basolateral and apical chambers were supplied with PneumaCult Ex Plus medium for 2 to 3 days, and then fed with PneumaCult-ALI media (#05001, StemCell) only in the basolateral chamber. After 4 weeks, the transepithelial electrical resistance (TEER) of the cultures was determined and only the cultures that had a TEER of over 1,000 Ω/cm^2^ were used.

### Plasmid constructs

#### NP1-expressing plasmids

Mammalian cell expression plasmid pCI-NP1^Flag^ and *E*. *coli* expression plasmid pET30a-NP1^His^ have been described previously [16,23]. pCI-NP1^m1-6^ were the derivatives of plasmid pCI-NP1^Flag^, expressing mutant NP1 with mutations shown in **Fig 13A**, respectively.

#### Bacterial expression plasmids

pET30a(+) (#69909, MilliporeSigma) was used to construct the following His-tagged protein-expressing plasmids: pET30a-Ku70^His^, pET30a-Ku80^His^, pET30a-Ku70-vWA^His^, pET30a-Ku70-Core^His^, pET30a-Ku70-SAP^His^, pET30a-RPA70^His^, pET30a-RPA32^His^, pET30a-RPA70-N-ter^His^, pET30a-RPA70-AB^His^, pET30a-RPA70-C-ter^His^, pET30a-RPA70-A^His^, pET30a-RPA70-B^His^, pET30a-HBoV1-NS1^His^, pET30a-MBP^His^, and pET30a-Ku70^Strep^.

pMBP-Ku70-β-barrel^His^ and pMBP-Ku70-CTR^His^ were constructed by inserting the ORF of the indicated proteins into pMAL-c5X-His (#P8114, New England Biolabs, MA, USA). pGEX-4T3 (#28954552, Cytiva Life Science, Marlborough, MA) was used to construct pGST-NP1, pGST-NP1^m1-6^, and pGST^Flag^ vectors.

#### pIHBoV1 plasmids

HBoV1 infectious clone plasmid pIHBoV1 and the NP1 knockout mutant pIHBoV1^ΔNP1^ have been described previously [35].

#### pTripZ plasmids

Two codon-optimized sequences of the ORFs of Ku70-β-barrel and RPA70-AB domains with tags of Strep and HA were synthesized and cloned into mammalian expression vector pcDNA3.1 for the plasmids, pcDNA-opt1/2-Ku70-β-barrel^Strep-HA^ and pcDNA-opt1/2-RPA70-AB^Strep-HA^, at Genscript (Piscataway, NJ). The pTripZKu70-β-barrel and pTripZRPA70-AB vectors were constructed by inserting Ku70-β-barrel and RPA70-AB encoding sequences into pTripZ-NS1^Strep-Flag^ [86] via the AgeI and MluI restriction sites. pTripZmCherry plasmid has been described previously [49].

### Protein expression and purification

The protein expression plasmids with the target cloned in pET30a(+), pMAL-c5X-His, and pGEX-4T3 were transformed into BL21/DE3 pLysS *E*.*coli* bacteria (#L1195, Promega, Madison, WI). The recombinant His-, MBP-, Strep-, and GST-tagged proteins were purified as previously described [23,49,87,88] or purified with Bio-Rad NGC Chromatography System (Bio-Rad Laboratories) using Bio-Scale Mini Nuvia IMAC Cartridges (#7800811, Bio-Rad, CA). The His-tagged NS1-70 was further purified by running through a HiLoad Superdex 200 pg column (#28989335, Cytiva) with Bio-Rad NGC Chromatography System at a flow rate of 0.5 ml/min as previously described [49].

### Plasmid DNA transfection

HEK293 cells were transfected by using the PEI MAX transfection reagent (#24765–2, Polyscience, Inc., Warrington, PA) as previously described [17]. An amount of 2 μg plasmid/well was used for 6-well plate and 4 μg plasmid was transfected for each 60-mm dish.

### Virus infection and quantification

Virus infection and collection were performed following previously published guidelines [34,36]. Briefly, HBoV1 was diluted with D-PBS (#21-031-CV, Corning, NY). And 100 μl diluent, which contains an amount of multiplicity of infection (MOI) of 100 DNase I-resistant viral particles (DRP), was applied to the apical chamber of each well of HAE-ALI cultures. After incubation at 37°C for 1 h, the chamber was washed twice with D-PBS. At indicated time points, 300 μl of D-PBS was added into the apical chamber of the infected HAE-ALI cultures and incubated at 37°C for 1 h, followed by collection of the inoculum, which was quantified for viral genome copies by qPCR as described previously [30].

### Lentivirus production and transduction

We produced lentiviruses according to the instructions of Addgene (http://www.addgene.org/tools/protocols/plko). The produced lentiviruses were concentrated, and transduction unit (TU) were titrated as previously described [89]. HEK293 cells were transduced at an MOI of ~5 TU/cell. To integrate the tet-on expression in HAE-ALI, proliferating HAE cells were seeded onto Transwell inserts (4.6 x 10^5^/insert) with PneumaCult- Ex Plus media (Stemcell) in both chambers. The cells were infected with lentivirus at an MOI of ~5 one day after seeding. At 2 days post-transduction, culture medium was switched to PneumaCult ALI medium, but were only supplied to the basolateral chamber to enable the differentiation of cell polarization at an ALI for ~4 weeks, as described for HAE-ALI cultures. Doxycycline was added to the basolateral medium at 2 μg/ml to induce the expression.

### Immunoprecipitation (IP) assay to identify the NP1-interacting proteins

HEK293 cells seeded on a 150-mm dish were co-transfected with pIHBoV1^ΔNP1^ and pCI-NP1^Flag^ plasmids at 1:1 ratio (12.5 μg: 12.5 μg) using PEI MAX transfection reagent to initiate HBoV1 replication. Cells were lysed at 48 h post-transfection by adding 3 ml of Lysis buffer [50 mM Tris pH7.5, 150 mM NaCl, 0.5% NP-40, 1 mM EDTA, 10% glycerol, 1x protease inhibitor cocktail (S8830, MilliporeSigma) and 1x phosphatase inhibitor (PhosStop; Roche, MillliporeSigma)] to the monolayer. Cells were incubated with lysis buffer for 15 min and transferred to tubes and passed through a 21G needle at least three times to facilitate lysis. The cell lysates were clarified by centrifugation at 16,000 *g* for 15 min. The clarified cell lysates were collected. 10% of the cell lysates were saved for input and stored at -80°C. The remains were equally distributed into 2 tubes (1.2 ml per tube) and incubated at 4°C for 4 h after adding 5 μg anti-Flag (#F1804, MilliporeSigma) or control IgG antibody (Mouse G3A1 Isotype control; # 5415S, Cell Signaling). The antigen/antibody complex were captured by adding 100 μl of protein G beads (50% slurry) and incubated for 2 h. The beads were washed with Lysis buffer (minus the protease inhibitors) five times. The proteins were eluted in 50 μl of 2 ×Laemmli loading buffer by boiling at 95°C for 5 min and resolved in a large format (15 x 18 cm; Whatman) SDS-10%PAGE gel and stained with Coomassie blue for 2 h followed by distaining until clear bands were observed. The unique bands in the anti-Flag group were excised, kept in 1.5-ml tubes and sent for in gel digestion and MS analysis at the Taplin Biological Mass Spectrometry Facility, Harvard Medical School.

### Co-Immunoprecipitation (Co-IP) assay

HEK293 cells were transfected with pCI-NP1^Flag^ and vector control. At 2 days post-transfection, the cells were washed twice with cold PBS and lysed with lysis buffer [50 mM Tris-HCl, pH 8.0, 150 mM NaCl, 1% NP-40, and Protease Inhibitor Cocktail (#S8830, SIGMAFAST, MilliporeSigma)] by constant agitation for 30 min at 4°C. Then, the cell lysates were treated with 250 units of Benzonase nuclease (#E1014-5KU, MilliporeSigma) and centrifugated at 12,000 g for 15 min at 4°C. The supernatant was collected and incubated with 40 μl of prewashed anti-Flag G1 affinity resin (GenScript, Piscataway, NJ) with rotation for ~4 h at 4°C. The beads were then pelleted down by centrifugation at 2,000 g for 3 min, followed by washing with Wash buffer (25 mM Tris-HCl, pH 8.0, 150 mM NaCl, 1% NP-40, and 1 mM EDTA) three times. The washed beads were mixed with 2 x Laemmli loading buffer and boiled at 95°C for 5 min to elute bound proteins for Western blotting.

### *In vitro* pull-down assay

Glutathione agarose resins (#16100, ThermoFisher, Waltham, MA) were prewashed and blocked with 3% BSA-PBS for 3 h. 4 μg of purified bait proteins, GST-NP1 and GST^Flag^, were immobilized on 30 μl of blocked Glutathione agarose in Binding buffer (25 mM Tris, pH 7.4, 150 mM NaCl, 1 mM EDTA, and 1% NP-40) at 4°C for 2 h with rotation. The purified prey proteins, Ku70/80^His^ heterodimer (#CT018-H07B, Sino Biological, Wayne, PA), Ku70^His^, Ku80^His^, Ku70-vWA^His^, Ku70-Core^His^, Ku70-SAP^His^, MBP-Ku70-β-barrel^His^, MBP-Ku70-CTR^His^, RPA70^His^, RPA32^His^, RPA70-N-ter^His^, RPA70-AB^His^, RPA70-C-ter^His^, RPA70-A^His^, or RPA70-B^His^ were then added to the mixture and incubated at 4°C for 3 h with rotation. The beads were washed 3 times with Washing buffer (25 mM Tris-HCl, pH 7.4, 150 mM NaCl, 0.5% NP-40, and 1 mM EDTA). The bound proteins were then eluted by boiling in 2 × Laemmli buffer at 95°C for 5 min and analyzed using Western blotting. Pull-down assay using Strep-tactin agarose (IBA Lifesciences, Germany) was performed in a similar manner. Briefly, 4 μg of the purified Ku70^Strep^ was first bound to the beads in binding buffer and then 4 μg of RPA70^His^ was incubated with the Strep-tactin agarose. MBP^His^ protein (4 μg) was used as a negative control. After washing three times with the Wash buffer, the beads were boiled for Western blotting.

In the *in vitro* pull-down assay, 3 units of benzonase was added to both the bait and prey protein at room temperature for 15 min to remove any DNA/RNA contaminations that might mediate the interactions.

### Western blotting

Western blotting was carried out as previously described [23]. Briefly, the cell lysates or protein samples were separated on SDS-PAGE gels and transferred onto a polyvinylidene difluoride (PVDF) membrane (#IPVH00010; MilliporeSigma). The transferred membrane was then blocked with 5% non-fat milk and incubated with indicated primary and secondary antibodies accordingly. Specific signals were visualized by enhanced chemiluminescence using a Fuji LAS4000 imaging system (Cytiva) or by a LI-COR Odyssey imaging system (LI-COR Corporate, Lincoln, NE).

### *In vivo* DNA replication assay

#### Low-molecular-weight (Hirt) DNA extraction

HAE-ALI cultures transduced with TripZmCherry, TripZRPA70AB, or TripZKu70-β-barrel lentivirus were infected with HBoV1. At 6 and 14 dpi, the infected HAE-ALI cultures were collected for Hirt DNA extraction [23]. HAE cells were dissociated from the Transwell inserts with Accutase (#AT104, Innovative Cell Technologies, Inc., San Diego, CA) at 37°C for 30 min and washed twice with PBS, followed with addition of Lysis buffer (10 mM Tris, pH 8.0, 10 mM EDTA, and 0.6% SDS) at room temperature for 15 min. Then, 5 M NaCl was added into the lysates to a final concentration of 1.5 M. After incubation overnight at 4°C, the cell lysates were centrifugated at 14,000 × rpm for 20 mins. Proteinase K was added to the supernatants at a final concentration of 1 mg/ml at 37°C for 1 h, followed by the DNA purification with a DNA gel extraction kit (#28706, Qiagen, Hilden, Germany) according to the manufacturer’s instructions.

#### Southern blotting

Southern blotting was performed according to our previously reported methods [15]. Briefly, Hirt DNA samples were resolved on 1% agarose gel, and the separated DNAs were transferred onto a supported nitrocellulose membrane (#1212590, GVS North America, ME) and probed with an [α-^32^P] dCTP-labeled probe of the HBoV1 *NSCap* gene [24]. HBoV1 DNA excised from SalI and XhoI-digested pIHBoV1 plasmid was used as the probe template. Hybridization signals were captured by using a storage phosphor screen and visualized on an Amersham Typhoon Biomolecular Imager (Cytiva). Signals were quantified using ImageQuant Tl (IQTL) 8.2 (Cytiva).

#### *In vitro* viral DNA replication assay

293ES cell extracts were prepared using a modified method as previously described [78,90,91]. Briefly, 293ES cells were cultured in ESF-SFM media (Expression Systems). When the cells grown in S-phase reached 2 x 10^6^ per ml, the cells were collected by centrifugation at 450 g for 15 min at 4°C. The medium was discarded, and the pooled cell pellets were washed twice with ice-cold PBS. After additional washes of two times in ice-cold Stillman’s hypotonic buffer [20 mM HEPES-KOH (pH 7.5), 5 mM KCl, 1.5 mM MgCl_2_, 1 mM DTT, and 1 mM PMSF] [90]. The pellet was then resuspended in ice-cold Stillman’s hypotonic buffer, and placed on ice for 10 min. The cells were homogenized slowly in a prechilled loose pestle Dounce homogenizer (PYREX type B; Corning, NY) with 15 strokes. The disrupted cells were kept on ice for a further 10 min, and 5 M KCl was added to a final concentration of 0.25 M. After incubation on ice for another 5 min, the mixture was centrifuged at 10,000 g for 15 min at 4°C. The supernatant was collected for preparation cytoplasmic extract (S100) and the pellet was pooled for the preparation of nuclear extract (S300).

#### Extraction of S100

The supernatant was centrifuged at 10,000 g for 30 min at 4°C and further centrifuged at 150,000 g at 4°C for 1 h. The resultant supernatant was collected and dialyzed in Slide-A-Lyze Dialysis Cassettes (#66810, ThermoFisher) at 4°C against Dialysis buffer [20 mM-HEPES-NaOH, pH 7.5, 25 mM KCl, 10% (v/v) glycerol, 1 mM DDT, and Protease Inhibitor Cocktail (#S8830, MilliporeSigma)]. The dialytic supernatant was centrifuged again at 10,000 g at 4°C for 30 min. The supernatant was aliquoted, frozen immediately in liquid nitrogen and stored at -80°C.

#### Extraction of S300

The nuclear pellet was resuspended in Hypotonic lysis buffer with slow addition of 2.5 M KCl to a final concentration of 0.25 M. The mixture was continuously and gently mixed for 30 min at 4°C, and further disrupted with a tight Dounce homogenizer (Corning) for 15 strokes. After pre-clarification at 12,000 g for 5 min, the supernatant was centrifuged at 300,000 g at 4°C for 1 h. The supernatant was aliquoted, frozen immediately in liquid nitrogen, and stored at -80°C.

#### *In vitro* viral DNA replication

The HBoV1 *in vitro* DNA replication was modified from the AAV *in vitro* replication assay as described previously [52,91]. The standard reaction mixture (50 μl) contains: 5 μl of 10 × reaction buffer (40 mM HEPES-KOH, pH 7.5; 7 mM MgCl_2_; 0.5 mM DTT), 2.5 μl of 20 × NTPs/dNTPs (4 mM ATP; 200 μM CTP/GTP/UTP; 100 μM dATP/dTTP/dGTP; 30 μM dCTP) (#R0441; #R0451; #R0461; #R0471; #R0141; #R0151; #R0161; #R0171, ThermoFisher), 40 mM phosphocreatine di(tris) salt (pH7.7) (#P1937, Sigma-Aldrich, MO), 2 μg of creatine phosphokinase (#C3755, MilliporeSigma), 200 μg of S100, 16 μg of S300, 200 ng of the HBoV1 duplex genome (SalI and XhoI digested pIHBoV1), 2 μg of the purified HBoV1 NS1-70, and 25 μCi of [α^-32P^] dCTP (#BLU013Z250UC, Perkin Elmer, MA). The reaction mixture was incubated at 37°C overnight. After adding 5 μl 10 × Stop buffer [5 mM EDTA, 0.5% (w/v) SDS] to stop the reaction, the mixture was digested with 1 μl proteinase K (10 mg/ml, #P2308, Sigma-Aldrich, MO) at 37°C for 1h. After purification with the Oligo Clean & Concentrator Kit (#D4061, Zymo Research, CA), samples were digested with 1 μl of DpnI (#R0176S, NEB, MA) to degrade the HBoV1 duplex genome (methylated bacterial DNA). The *in vitro* replicated viral DNA was then separated on 1% agarose gel at 120 V for 150 min, running with Tris-acetate-EDTA (TAE) buffer. The gel was washed twice with deionized water and dried on a filter paper in a gel dryer (Heto Dry GD-1, Heto Lab Equipment, Denmark). The signals were captured by exposing the dried gel to a storage phosphor screen overnight and visualized on the Amersham Typhoon Biomolecular Imager (Cytiva). Signals were quantified using ImageQuant Tl (IQTL) 8.2 (Cytiva).

### Biolayer interferometry (BLI) assay

BLI kinetics analysis was performed using a BLItz biolayer interferometry system (ForteBio/Sartorius, Bohemia, NY) as previously reported [92]. Briefly, the biosensors were first hydrated with Kinetic buffer (50 mM Tris-HCl, pH 7.4, and 150 mM NaCl) for 10 min, then different concentration of purified Ku70^His^, MBP-Ku70-β-barrel^His^, RPA70^His^, or RPA70-AB^His^ in kinetic buffer were mounted on Ni-NTA biosensors (#18–5101, ForteBio/Sartorius). After dipping into the Kinetic buffer to generate the baseline, the Ni-NTA biosensors were dipped into binding buffer containing 4 uM GST-NP1 or control GST^Flag^ to determine the binding parameters. The biosensor was dipped into the Kinetic buffer to finish a final dissociation step. The K_ass_ (association rate constant) and K_diss_ (dissociation rate constant) were determined using BLItz Pro software (ForteBio/Sartorius), assays were repeated at least three times for the calculation of the K_D_ (mean ± standard deviation).

### Antibodies used in the study

#### First antibodies

The following first antibodies were purchased: rabbit anti-HNRNPU (#A3917), rabbit anti-PUF60 (#A6709), rabbit anti-YBX1(#A7704), rabbit anti-DDX9 (#A4563), rabbit anti-NCL (#A5904), rabbit anti-ILF3 (#A8186), rabbit anti-DKC1(#A1862), rabbit anti-TOP2A (#A16440), rabbit anti-RPA70 (#A0990) and rabbit anti-STAU1 (#A4131) from Abclonal (Woburn, MA); mouse anti-Ku70 (#SC-17789) from Santa Cruz (Dallas, TX); rabbit anti-Ku80 (#2180S) and rabbit anti-His (#12698S) from Cell Signaling; mouse anti-Flag (#200-301-B13) from Rockland (Limerick, PA); mouse anti-β-actin (#A5441) from MilliporeSigma; anti-GST (#SC-138) from Santa Cruz (Dallas, TX); rabbit anti-Strep-tag II (#ab183907) from Abcam (Waltham, MA);

#### Secondary antibodies

HRP-conjugated anti-rabbit IgG (#A0545) and HRP-conjugated anti-mouse IgG (#A4416) were purchased from MilliporeSigma; DyLight 800 Conjugate anti-rabbit IgG (#5151S) and DyLight 800 Conjugate anti-mouse IgG (#5257S) from Cell Signaling.

### Statistical analysis

Statistical analysis was performed by using GraphPad Prism 9, and error bars show means and standard deviations. P values of statistical significance were determined by using Student’s t test. ****, P<0.0001; ***, P<0.001; **, P<0.01; and *, P<0.05 were regarded as statistically significant and n.s. regarded as statistically no significance.

## Supporting information

S1 Fig *In vitro* pulldown assay of NP1 and Ku70 in the presence of ethidium bromide. 4 μg of purified GST-NP1 and GST protein (a negative control) were used as baits to pull down 4 μg of Ku70^His^ prey protein in the binding buffer containing 0.2 mg/ml ethidium bromide [93], using Glutathione agarose. ~0.4 μg of prey protein was loaded as inputs. The pulldown protein was analyzed by Western blotting using anti-His for Ku70^His^ (**A**) or using anti-GST for GST-NP1 and GST (as controls) (**B**). M, protein size ladder marker.
(TIF)

S2 Fig NP1^Strep^ directly interacts with Ku70^Strep^. **(A) Purification of HBoV1 NP1**^**Strep**^. NP1^Strep^ protein was purified using Strep-Tactin agarose. Purity was analyzed on an SDS-(4–20%) PAGE gel stained with Coomassie brilliant blue. **(B-D) *In vitro* pull-down assay.** 4 μg of purified NP1^Strep^ was used as a bait to pull down 4 μg of the prey protein, purified Ku70^His^ (B) or His-tagged Ku70/80 heterodimer (C), using Strep-Tactin agarose. 4 μg of MBP^Strep^ served as a negative control. Proteins pulled down by the Strep-Tactin agarose were separated on SDS-PAGE gel and blotted with anti-His for Ku70^His^ (B) and for Ku70/80 heterodimer (C). ~0.4 μg of prey proteins were used as an input. 4 μg of MBP^Strep^ protein served as a negative control and analyzed by Western blotting using anti-Strep (D).
(TIF)

S3 Fig NP1 binding of Ku70 does not affect the stability of Ku70. **(A) Protein controls.** 4 μg of purified Ku70^His^ and GST-NP1 proteins were analyzed on SDS-(4–20%)PAGE gel stained with Coomassie brilliant blue. **(B) *In vitro* pulldown assay of GST-NP1 and Ku70.** 4 μg of purified Ku70^His^ and GST-NP1 proteins were added into 300 μl binding buffer with addition of 30 μl pre-washed glutathione agarose. After incubation at 4°C for 4 h or overnight, the glutathione agarose were washed three times with wash buffer and pelleted by centrifugation at 3, 000 g for 1 min before addition of 1 × Laemmli sample buffer. The samples were boiled at 95°C for 5 min and separated on SDS-(4–20%)PAGE gel, followed by Coomassie brilliant blue staining. M, protein size ladder marker. O/N, overnight.
(TIF)

S4 Fig Detection of Ku70 and Ku80 in HAE-ALI cultures during the course of HBoV1 infection. HAE-ALI cultures were infected with HBoV1 at an MOI of ~100 or mock infected. At 7 and 14 dpi, the cells were collected, boiled in Laemmli loading buffer, and separated on SDS-(4–20%)PAGE gel, followed by Western blotting using anti-Ku70 (A) and anti-Ku80 (B) antibodies, respectively. The signals were visualized by a LI-COR Odyssey imaging system. M, protein size ladder marker.
(TIF)

S1 Table Mass spectrometry identification of each protein band (Bands 1–4) and section (Sections 1–2) excised from the gel shown in Fig 1B. Numbers of the Unique/Total peptides of each identified protein are shown with the identification number and name (Reference), gene, and molecular weight (MW).
(XLS)
