# Supplementary figures and images for: The small nonstructural protein NP1 of human bocavirus 1 directly interacts with Ku70 and RPA70 and facilitates viral DNA replication
Source: PLoS Pathog. 2022 Jun 2;18(6):e1010578. doi: 10.1371/journal.ppat.1010578 (PMC9197078; doi:10.1371/journal.ppat.1010578)

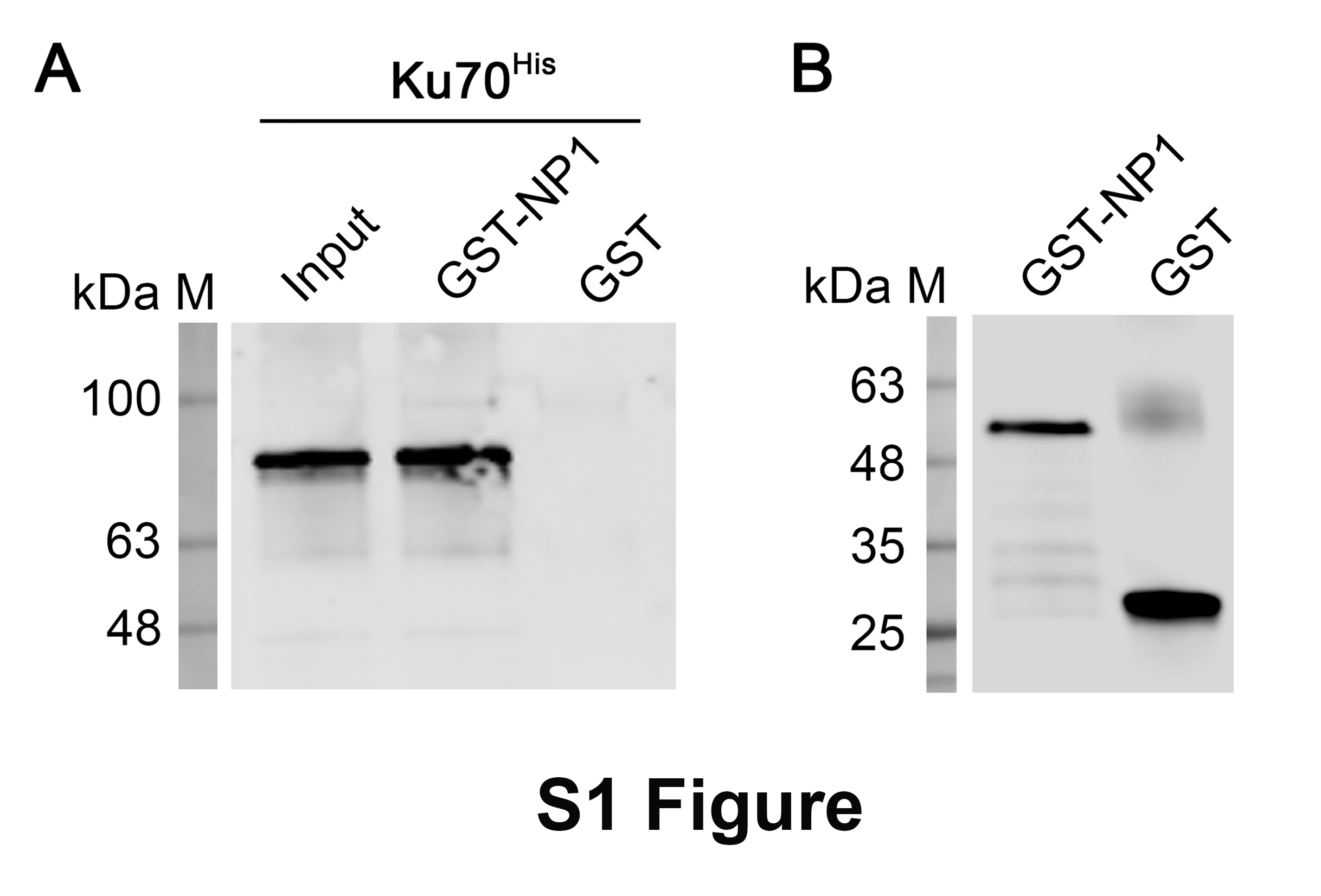

Supplement: S1 Fig — 4 μg of purified GST-NP1 and GST protein (a negative control) were used as baits to pull down 4 μg of Ku70His prey protein in the binding buffer containing 0.2 mg/ml ethidium bromide [93], using Glutathione agarose. ~0.4 μg of prey protein was loaded as inputs. The pulldown protein was analyzed by Western blotting using anti-His for Ku70His (A) or using anti-GST for GST-NP1 and GST (as controls) (B). M, protein size ladder marker. (TIF) [file ppat.1010578.s001.tif]

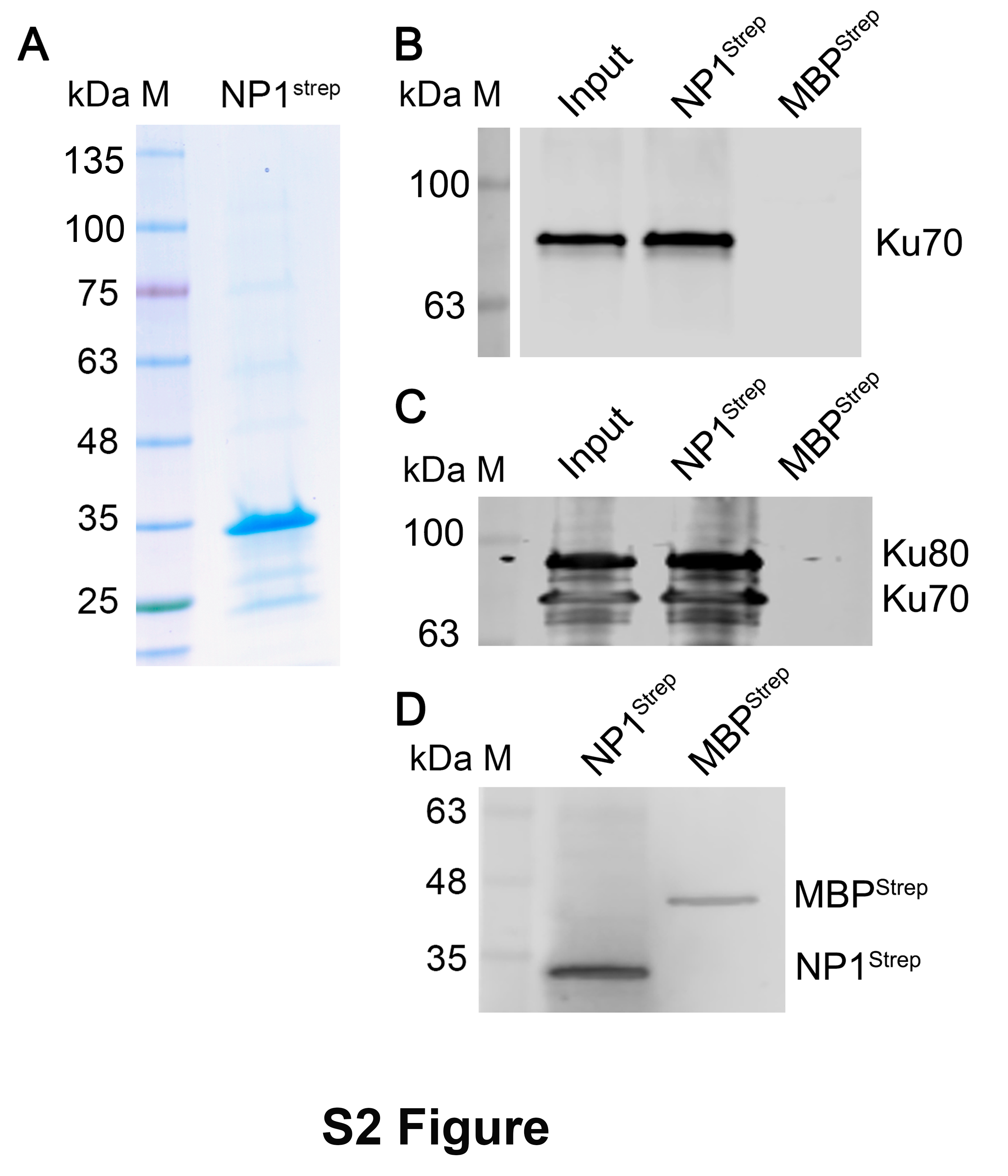

Supplement: S2 Fig — (A) Purification of HBoV1 NP1Strep. NP1Strep protein was purified using Strep-Tactin agarose. Purity was analyzed on an SDS-(4–20%) PAGE gel stained with Coomassie brilliant blue. (B-D) In vitro pull-down assay. 4 μg of purified NP1Strep was used as a bait to pull down 4 μg of the prey protein, purified Ku70His (B) or His-tagged Ku70/80 heterodimer (C), using Strep-Tactin agarose. 4 μg of MBPStrep served as a negative control. Proteins pulled down by the Strep-Tactin agarose were separated on SDS-PAGE gel and blotted with anti-His for Ku70His (B) and for Ku70/80 heterodimer (C). ~0.4 μg of prey proteins were used as an input. 4 μg of MBPStrep protein served as a negative control and analyzed by Western blotting using anti-Strep (D). (TIF) [file ppat.1010578.s002.tif]

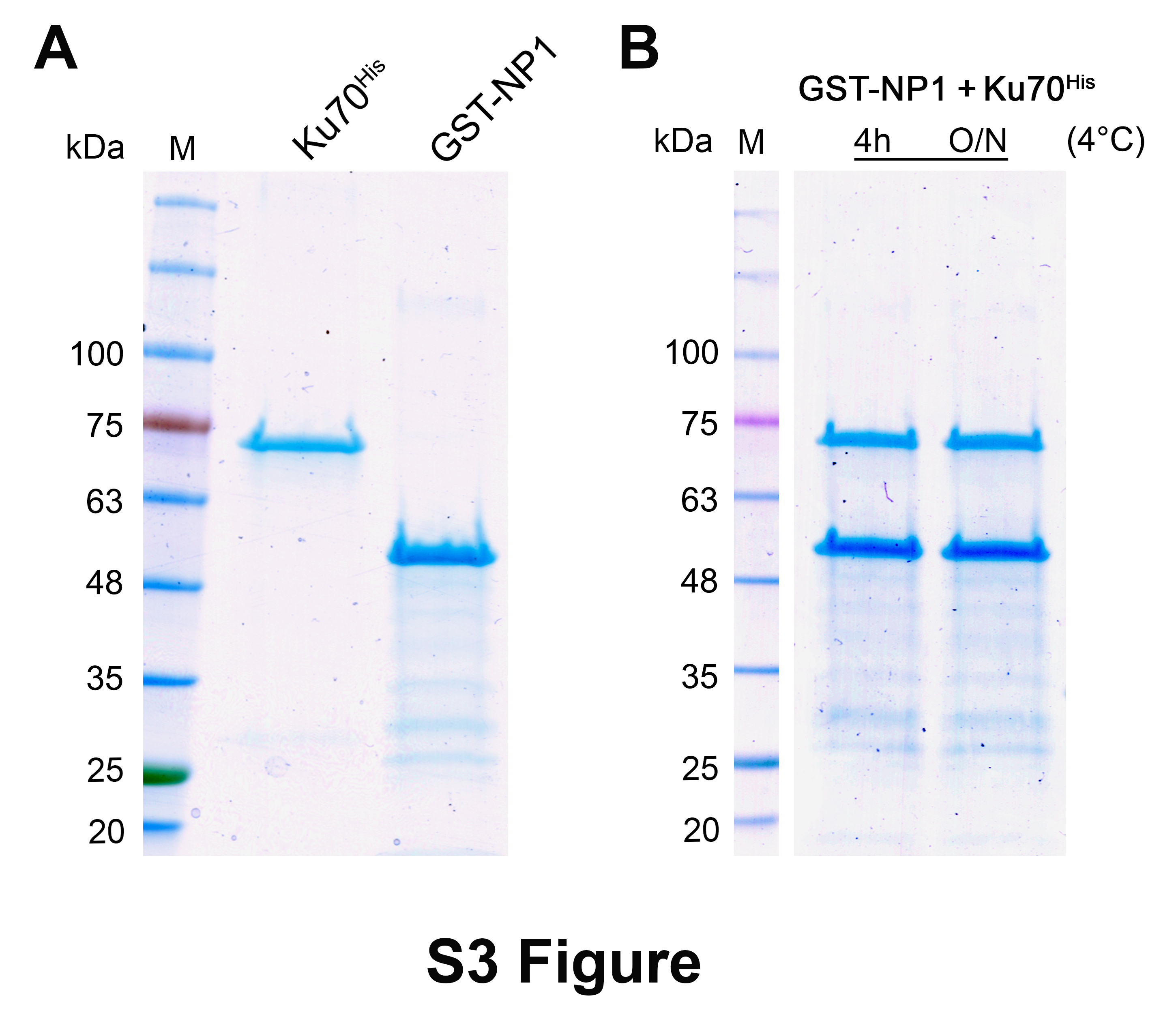

Supplement: S3 Fig — (A) Protein controls. 4 μg of purified Ku70His and GST-NP1 proteins were analyzed on SDS-(4–20%)PAGE gel stained with Coomassie brilliant blue. (B) In vitro pulldown assay of GST-NP1 and Ku70. 4 μg of purified Ku70His and GST-NP1 proteins were added into 300 μl binding buffer with addition of 30 μl pre-washed glutathione agarose. After incubation at 4°C for 4 h or overnight, the glutathione agarose were washed three times with wash buffer and pelleted by centrifugation at 3, 000 g for 1 min before addition of 1 × Laemmli sample buffer. The samples were boiled at 95°C for 5 min and separated on SDS-(4–20%)PAGE gel, followed by Coomassie brilliant blue staining. M, protein size ladder marker. O/N, overnight. (TIF) [file ppat.1010578.s003.tif]

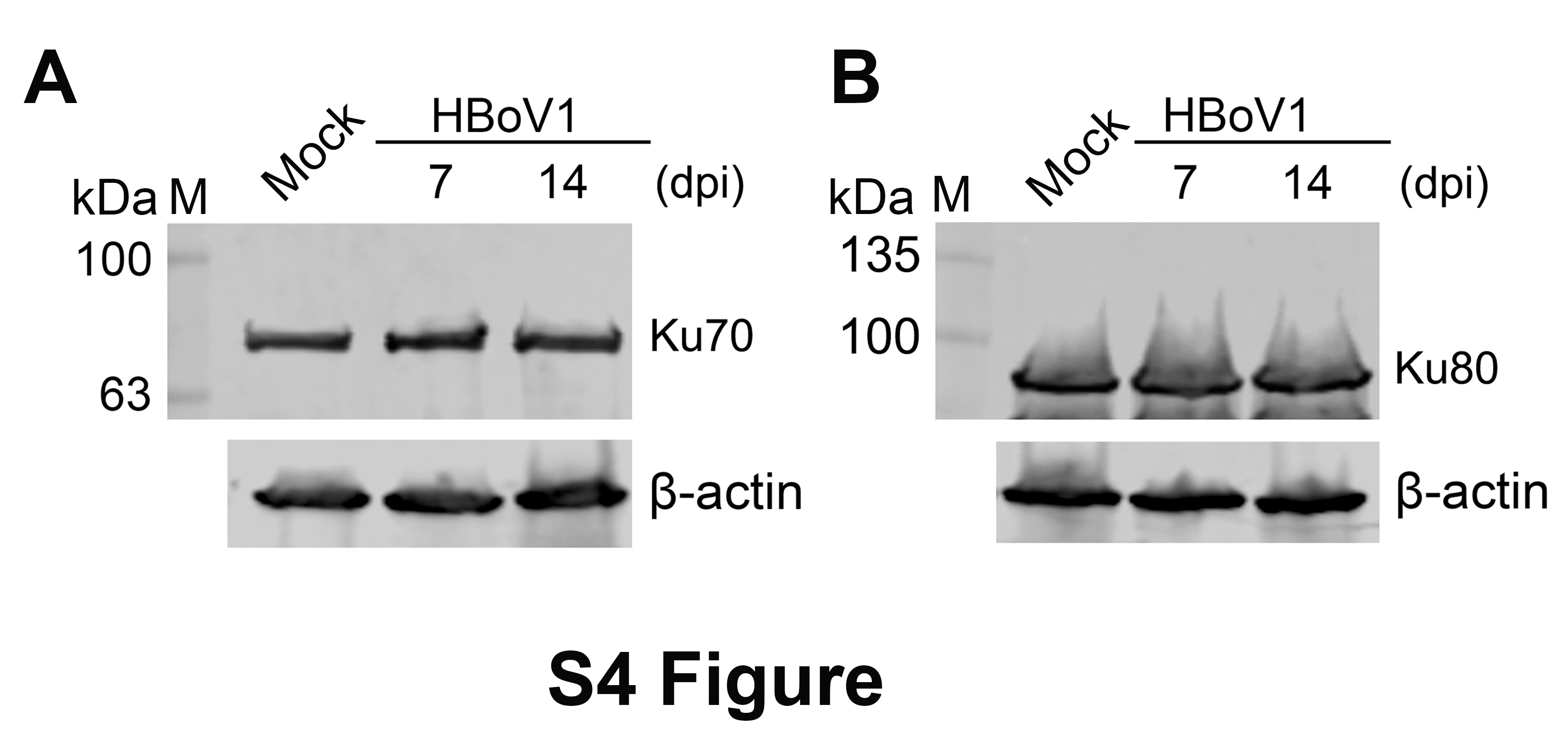

Supplement: S4 Fig — HAE-ALI cultures were infected with HBoV1 at an MOI of ~100 or mock infected. At 7 and 14 dpi, the cells were collected, boiled in Laemmli loading buffer, and separated on SDS-(4–20%)PAGE gel, followed by Western blotting using anti-Ku70 (A) and anti-Ku80 (B) antibodies, respectively. The signals were visualized by a LI-COR Odyssey imaging system. M, protein size ladder marker. (TIF) [file ppat.1010578.s004.tif]
